# Transcription factor StABI5-like 1 binding to the FLOWERING LOCUS T homologs promotes early maturity in potato

**DOI:** 10.1093/plphys/kiac098

**Published:** 2022-03-08

**Authors:** Shenglin Jing, Xiaomeng Sun, Liu Yu, Enshuang Wang, Zhengnan Cheng, Huimin Liu, Peng Jiang, Jun Qin, Shahnewaz Begum, Botao Song

**Affiliations:** Key Laboratory of Horticultural Plant Biology (HZAU), Ministry of Education, Wuhan, Hubei 430070, China; Key Laboratory of Potato Biology and Biotechnology (HZAU), Ministry of Agriculture and Rural Affairs, Wuhan, Hubei 430070, China; Potato Engineering and Technology Research Center of Hubei Province, Huazhong Agricultural University, Wuhan, 430070, China; College of Horticulture and Forestry Science, Huazhong Agricultural University, Wuhan, Hubei, 430070, China; Key Laboratory of Horticultural Plant Biology (HZAU), Ministry of Education, Wuhan, Hubei 430070, China; Key Laboratory of Potato Biology and Biotechnology (HZAU), Ministry of Agriculture and Rural Affairs, Wuhan, Hubei 430070, China; Potato Engineering and Technology Research Center of Hubei Province, Huazhong Agricultural University, Wuhan, 430070, China; College of Horticulture and Forestry Science, Huazhong Agricultural University, Wuhan, Hubei, 430070, China; Key Laboratory of Horticultural Plant Biology (HZAU), Ministry of Education, Wuhan, Hubei 430070, China; Key Laboratory of Potato Biology and Biotechnology (HZAU), Ministry of Agriculture and Rural Affairs, Wuhan, Hubei 430070, China; Potato Engineering and Technology Research Center of Hubei Province, Huazhong Agricultural University, Wuhan, 430070, China; College of Horticulture and Forestry Science, Huazhong Agricultural University, Wuhan, Hubei, 430070, China; Key Laboratory of Horticultural Plant Biology (HZAU), Ministry of Education, Wuhan, Hubei 430070, China; Key Laboratory of Potato Biology and Biotechnology (HZAU), Ministry of Agriculture and Rural Affairs, Wuhan, Hubei 430070, China; Potato Engineering and Technology Research Center of Hubei Province, Huazhong Agricultural University, Wuhan, 430070, China; College of Horticulture and Forestry Science, Huazhong Agricultural University, Wuhan, Hubei, 430070, China; Key Laboratory of Horticultural Plant Biology (HZAU), Ministry of Education, Wuhan, Hubei 430070, China; Key Laboratory of Potato Biology and Biotechnology (HZAU), Ministry of Agriculture and Rural Affairs, Wuhan, Hubei 430070, China; Potato Engineering and Technology Research Center of Hubei Province, Huazhong Agricultural University, Wuhan, 430070, China; College of Horticulture and Forestry Science, Huazhong Agricultural University, Wuhan, Hubei, 430070, China; Key Laboratory of Horticultural Plant Biology (HZAU), Ministry of Education, Wuhan, Hubei 430070, China; Key Laboratory of Potato Biology and Biotechnology (HZAU), Ministry of Agriculture and Rural Affairs, Wuhan, Hubei 430070, China; Potato Engineering and Technology Research Center of Hubei Province, Huazhong Agricultural University, Wuhan, 430070, China; Key Laboratory of Horticultural Plant Biology (HZAU), Ministry of Education, Wuhan, Hubei 430070, China; Key Laboratory of Potato Biology and Biotechnology (HZAU), Ministry of Agriculture and Rural Affairs, Wuhan, Hubei 430070, China; Potato Engineering and Technology Research Center of Hubei Province, Huazhong Agricultural University, Wuhan, 430070, China; College of Horticulture and Forestry Science, Huazhong Agricultural University, Wuhan, Hubei, 430070, China; Key Laboratory of Horticultural Plant Biology (HZAU), Ministry of Education, Wuhan, Hubei 430070, China; Key Laboratory of Potato Biology and Biotechnology (HZAU), Ministry of Agriculture and Rural Affairs, Wuhan, Hubei 430070, China; Potato Engineering and Technology Research Center of Hubei Province, Huazhong Agricultural University, Wuhan, 430070, China; College of Horticulture and Forestry Science, Huazhong Agricultural University, Wuhan, Hubei, 430070, China; Key Laboratory of Horticultural Plant Biology (HZAU), Ministry of Education, Wuhan, Hubei 430070, China; Key Laboratory of Potato Biology and Biotechnology (HZAU), Ministry of Agriculture and Rural Affairs, Wuhan, Hubei 430070, China; Potato Engineering and Technology Research Center of Hubei Province, Huazhong Agricultural University, Wuhan, 430070, China; College of Horticulture and Forestry Science, Huazhong Agricultural University, Wuhan, Hubei, 430070, China; Key Laboratory of Horticultural Plant Biology (HZAU), Ministry of Education, Wuhan, Hubei 430070, China; Key Laboratory of Potato Biology and Biotechnology (HZAU), Ministry of Agriculture and Rural Affairs, Wuhan, Hubei 430070, China; Potato Engineering and Technology Research Center of Hubei Province, Huazhong Agricultural University, Wuhan, 430070, China; College of Horticulture and Forestry Science, Huazhong Agricultural University, Wuhan, Hubei, 430070, China

## Abstract

Potato (*Solanum tuberosum* L.) maturity involves several important traits, including the onset of tuberization, flowering, leaf senescence, and the length of the plant life cycle. The timing of flowering and tuberization in potato is mediated by seasonal fluctuations in photoperiod and is thought to be separately controlled by the *FLOWERING LOCUS T-like* (*FT-like*) genes *SELF-PRUNING 3D* (*StSP3D*) and *SELF-PRUNING 6A* (*StSP6A*). However, the biological relationship between these morphological transitions that occur almost synchronously remains unknown. Here, we show that StABI5-like 1 (StABL1), a transcription factor central to abscisic acid (ABA) signaling, is a binding partner of StSP3D and StSP6A, forming an alternative florigen activation complex and alternative tuberigen activation complex in a 14-3-3-dependent manner. Overexpression of *StABL1* results in the early initiation of flowering and tuberization as well as a short life cycle. Using genome-wide chromatin immunoprecipitation sequencing and RNA-sequencing, we demonstrate that *AGAMOUS-like* and *GA 2-oxidase 1* genes are regulated by StABL1. Phytohormone profiling indicates an altered gibberellic acid (GA) metabolism and that *StABL1*-overexpressing plants are insensitive to the inhibitory effect of GA with respect to tuberization. Collectively, our results suggest that StABL1 functions with *FT-like* genes to promote flowering and tuberization and consequently life cycle length in potato, providing insight into the pleiotropic functioning of the *FT* gene.

## Teaser

StABI5-like 1, a transcription factor central to ABA signaling, functions with FLOWERING LOCUS T-like proteins to promote early flowering, tuberization, and life cycle length in potato.

## Introduction

Potato (*Solanum tuberosum* L.), a staple food of critical importance in terms of food security, is cultivated for its underground storage organs or tubers, which accumulate large amounts of starch and vitamin C. Potato tuberization is used as a model system to study the formation of specialized vegetative organs in geophytic species, which are characterized by a dual reproduction system where both sexual and vegetative reproduction are adopted to survive under fluctuating photoperiod or adverse conditions (Khosa et al., 2021; Zierer et al., 2021). Storage organ formation and flower induction are regulated by similar molecular cascades, in which FLOWERING LOCUS T (FT) proteins, members of the phosphatidylethanolamine-binding protein (*PEBP*) gene family, are major players (Navarro et al., 2011, 2015). Long-day (LD) flowering and short-day (SD) tuberization in potato are controlled by two different FT paralogs, called florigen StSP3D and tuberigen StSP6A, respectively (Navarro et al., 2011). Although other mobile signaling molecules controlling potato tuberization have been reported, such as *miR156*, *miR172*, and *StBEL5* (Banerjee et al., 2006; Martin et al., 2009; Bhogale et al., 2014), the mode of action of these molecules and environmental effects on tuber formation converge on *StSP6A* expression (Navarro et al., 2015; Sharma et al., 2016; Lehretz et al., 2019).

Under inductive SD photoperiod, StSP6A is synthesized in the leaves and transported to the stolons for tuber induction. Under LDs, the expression of *StSP6A* is blocked by another FT homolog, StSP5G, which is directly activated by CONSTANS-LIKE 1 (StCOL1). Natural truncated alleles of *CYCLING DOF FACTOR 1* (*StCDF1*), the potato *earliness* locus, evade proteasome-dependent degradation by StGI/StFKF1 under LDs, and stabilized StCDF1 repress *StCOL1* expression under LDs; therefore, *StSP5G* is not induced, thus allowing tuberization (Kloosterman et al., 2013). Genetic knockdown of *StCOL1* or *StSP5G* in the photoperiod-sensitive genotype *Andigena* activates both *StSP3D* and *StSP6A*, leading to early flowering and tuberization under noninductive LDs (Abelenda et al., 2016). In potato, the induced expression of *StSP6A* occurs alongside the induction of senescence (Lehretz et al., 2019). The onset of tuberization, flowering, leaf senescence, and life cycle length are considered as important traits of potato maturity or maturity syndrome, and are used to score maturation in potato (Visker et al., 2003). However, the biological relationship among these developmental changes that occur almost synchronously remains largely unknown.

FT associates with a bZIP (basic leucine zipper) transcription factor (TF) flowering locus D (FD), bridged by a 14-3-3 protein via S/TAP motif in the C-terminus of FD, to form a complex called florigen activation complex (FAC) in the shoot apical meristem, allowing fine-tuning of downstream *SUPPRESSOR OF OVEREXPRESSION OF CO 1* (*SOC1*), *FRUITFUL* (*FUL*), and *APETALA1* genes (Abe et al., 2005; Wigge et al., 2005; Taoka et al., 2011). The class II TEOSINTE BRANCHED1/CYCLOIDEA/PCF (TCP) TF BRC1 also functions with FT independent of FAC to delay flowering of the developing axillary shoot in *Arabidopsis thaliana* (Niwa et al., 2013). Evidence from Arabidopsis also suggest that FT can function independent of FD and FDP (FD paralog), and genetic redundancy exists among group A bZIP TFs (Romera-Branchat et al., 2020), which consist of FD and FDP, and another subgroup encoding the highly related proteins AREB/ABF/ABI5 (Droge-Laser et al., 2018), which are core TFs in abscisic acid (ABA) signaling (Yoshida et al., 2015). Consistently, independent studies have found that the flower-inducing role of *PEBP* genes might be derived from an ancient role in ABA responses (Xi et al., 2010; Karlgren et al., 2011; Romera-Branchat et al., 2020). These data suggest that FT is essentially pleiotropic.

Analogous molecular modules in tuberization are reported in potato. StSP6A together with StFDL1 (FD homolog in potato) form a tuberigen activation complex (TAC) to induce tuberization in a 14-3-3-dependent manner (Teo et al., 2017). StCEN suppresses tuberization by directly antagonizing the function of StSP6A in TAC (Zhang et al., 2020). StBRC1b interacts with StSP6A to block its inducing activity in aerial axillary meristems (Michael et al., 2021). Moreover, evidence has shown that StSP6A binds and inactivates the sugar transporter StSWEET11 (sugar will eventually be exported transporters [SWEET]), thus blocking sugar leakage to enhance its symplastic transport (Abelenda et al., 2019). The arrival of the StSP6A signal to the stolon subapical region also triggers the local expression of the tuberization marker *StGA2ox1*, which blocks gibberellic acid (GA) activity (Kloosterman et al., 2007; Navarro et al., 2011). GA is the best recognized phytohormone that inhibits tuber formation by preventing cortical microtubule reorientation (Rodriguez-Falcon et al., 2006).

Despite growing knowledge of the function of protein complexes related to *FT* genes in potato flowering and tuberization, the molecular mode of action of *FT-like* genes in synchronizing potato maturity traits, including the onset of tuberization, flowering, and leaf senescence, as well as the length of plant life cycle, remains unknown.

In this study, we characterize StABI5-like 1 (StABL1), a member of the AREB/ABF/ABI5 subfamily (Liu et al., 2019), as a component to form alternative TAC (aTAC) and alternative FAC (aFAC) in potato, which are involved in flowering, tuberization, and life cycle length control by modulating ABA and GA responses. Our findings suggest that StABL1 binding to FT homologs promotes early maturity in potato, so exploring the natural variants or creating new alleles of *StABL1* using genome editing may promote the breeding of potato varieties suited to different geographical environments and harvest times.

## Results

### Characterization of StABL1 as an FT homolog binding partner

To explore whether other bZIP TFs expressed in stolons potentially form aTACs, amino acid sequences of StFD, StFDL1, and OsFDs were used as queries against the Spud DB (http://spuddb.uga.edu/) by protein–protein BLAST. StABL1 was identified with an S/TAP motif essential for 14-3-3 binding, and phylogenetic analysis showed that *StABL1* belongs to a subgroup of group A bZIPs encoding TFs involved in core ABA signaling (Supplemental Figure S1, A and B). Subcellular localization analysis using green fluorescent protein (GFP)-fused StABL1 and red fluorescent protein (RFP)-fused StSP6A indicated that they were colocalized in the nucleus in *Nicotiana benthamiana* pavement cells (Supplemental Figure S2A). Expression analysis revealed that *StABL1* was ubiquitously expressed in most of the tissues but was slightly higher in roots and developing stolons (Supplemental Figure S2, B–F). The protein sequence, subcellular localization, and expression profile analyses make StABL1 a potential component of aTAC.

**Figure 1:**
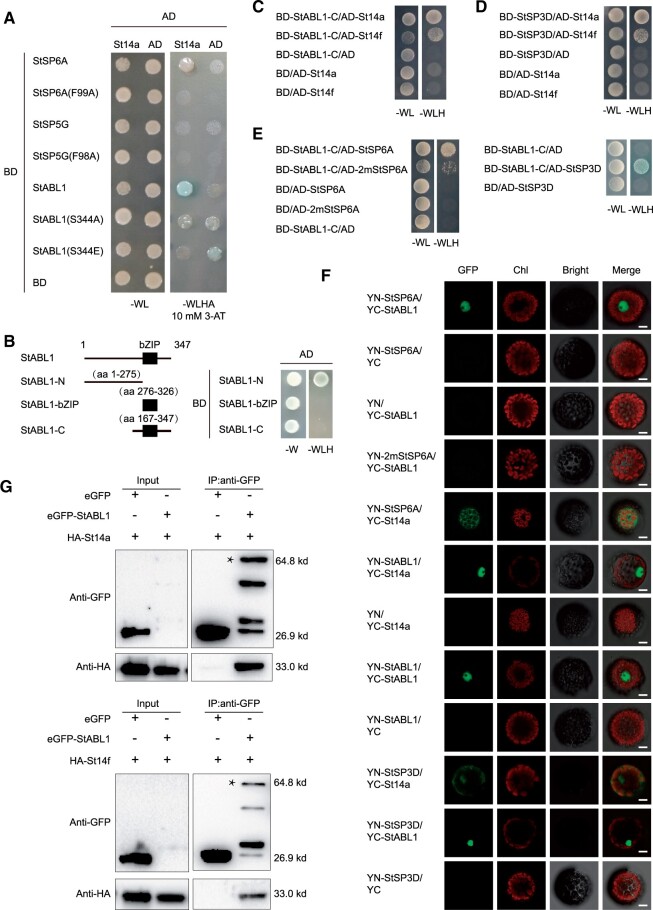
Interaction between StABL1 and FT-like paralogs. A, Interactions between FT and StABL1 with St14a in Y2H assays. StSP6A mutant F99A and StSP5G mutant F98A were tested; Alanine substitution (S344A) and phosphomimic mutation (S344E) on S/TAP motifs of StABL1 were also tested. −WL, medium without tryptophan and leucine; −WLHA, medium without tryptophan, leucine, histidine, and adenine. BD (pGBKT7) and AD (pGADT7) are the bait and prey vectors, respectively. 3-aminotriazole. B, Self-activation test of domain of StABL1 and its truncated fragments. StABL1-N (N-terminal of StABL1), StABL1-bZIP (bZIP fragment of StABL1), StABL1-C (C-terminal of StABL1). C, Y2H interaction analysis of StABL1-C and St14-3-3s. −WLH (medium without tryptophan, leucine, and histidine). D, Y2H interaction analysis of StSP3D with and St14-3-3s. E, Interactions between StSP6A and its mutant (2mStSP6A: R60K/P92L mutation), and StSP3D with StABL1-C in Y2H assays. F, BiFC analysis of the interaction among StSP6A, 2mStSP6A, StSP3D, StABL1, and St14a in E3 protoplasts. Chl, Chloroplast auto-fluorescence; YN, nYFP; YC; cYFP. Scale bar: 10 µm. G, Interaction between StABL1 and St14-3-3s in the Co-IP assays. The proteins were extracted from young coinjected leaves of *N. benthamiana* and immunoprecipitated by anti-GFP agarose beads. Gel blots were probed with anti-HA or anti-GFP antibody. The asterisk indicates the specific eGFP-StABL1 band. These nonspecific bands represent breakdown products resulting from protein turnover during IP.

To check whether StABL1 is involved in aTAC, we tested its interaction with potato 14-3-3 proteins (St14s) and StSP6A. First, we found that both StABL1 and StSP6A can interact with St14a in a yeast two-hybrid (Y2H) system, but mutated StSP6A (F99A) and StSP5G cannot (Figure 1A), and the C-terminus of StABL1 expressed in yeast without auto-activation was sufficient to interact with St14s (Figure 1, B and C). Second, we conducted bimolecular fluorescence complementation (BiFC) assays with StABL1 fused to the N-terminus of YFP (YN-StABL1) and St14a fused to the C-terminus of YFP (YC-St14a). When the combination containing both YN-StABL1 and YC-St14a constructs was transfected into the protoplasts isolated from E-potato 3 (E3) leaves, a strong fluorescent signal was observed in the nucleus (Figure 1F). Third, we performed co-immunoprecipitation (Co-IP) assays to determine these interactions in vivo. Constructs driving the expression of GFP: StABL1 or GFP were coinfiltrated in Nicotiana leaves with constructs expressing St14s: HA. The results showed that St14s: HA could be detected from the immunoprecipitated proteins of the GFP: StABL1-expressing leaves (Figure 1G), and similar results were obtained when GFP: StABL1 was immunoprecipitated (Supplemental Figure S2G), indicating that StABL1 and St14s can interact with each other in vivo. To further demonstrate the interactions between StABL1 and StSP6A, we used Y2H assays to test their interaction via yeast endogenous 14-3-3, and found that StABL1 and StSP6A can interact with each other, whereas interactions were much weaker for 2mStSP6A (mutated StSP6A: R60K/P92L) (Figure 1E; Supplemental Figure S1C). Then, the BiFC assay also confirmed their interaction in the nucleus, while StSP6A interacted with St14a mainly in the cytoplasm (Figure 1F). Altogether, these results indicate that StSP6A can interact with StABL1 via St14s mediators.

### Overexpression of *StABL1* causes early flowering, tuberization, and a short life cycle

To study the function of *StABL1*, we generated RNA interference lines (*RNAi-StABL1*), and two lines (RNAi-StABL1–32 and RNAi-StABL1–33) were selected for further study because the average percentages of downregulation were of 98% and 99% with RNAi-StABL1–32 and RNAi-StABL1–33, respectively (Supplemental Figure S3A). And GFP-fused *StABL1*-overexpressing transgenic lines (*OE-StABL1*) with *StABL1* driven by the cauliflower mosaic virus 35S promoter (Supplemental Figure S3B). Two lines (OE-StABL1–10 and OE-StABL1–13) were selected for further study because their proteins were highly expressed based on western blot and fluorescent signals (Supplemental Figure S3, C and D). As *StABL1* belongs to the *AREB/ABF/ABI5* subfamily and has a close relationship with *AtABI5* and *StABI5*, dark-induced leaf yellowing and stomatal movement, two well-recognized ABA-regulated process, were tested first. The results showed that dark-induced leaf yellowing was accelerated, and *StABL1*-overexpressing lines were more sensitive to ABA-induced stomatal closure than wild-type (WT) E3 (Supplemental Figure S3, E and F), indicating that *StABL1* is involved in ABA signaling in potato.

**Figure 2:**
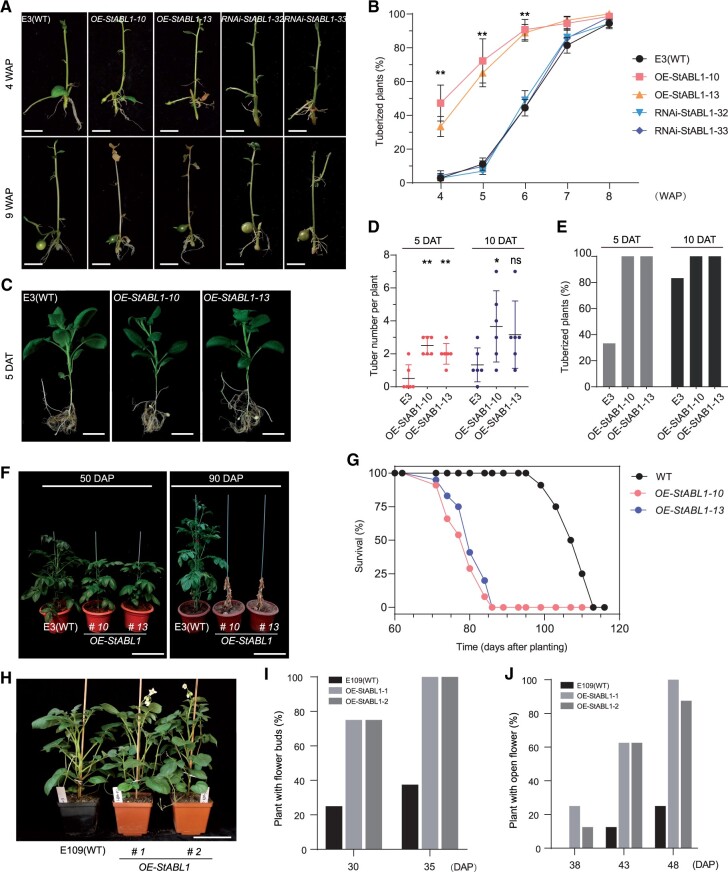
Tuberization, maturity, and flowering of *StABL1* transformants. A, The tuberization phenotype of representative E3, *RNAi-StABL1*, and *StABL1*-overexpressing (*StABL1ox*) transgenic plants in vitro 4 and 9 WAP, respectively. Scale bar: 1.5 cm. B, Percentage of in vitro cultured transgenic plants with micro-tubers. Data are presented as mean ± se. Data were obtained from 72 plants for each genotype. C, The tuberization phenotype of representative soil-grown WT E3 and *StABL1ox* transgenic plants at 5 DAT to SDs. Scale bar: 5 cm. D, Tuber number per plant of soil-grown WT E3 and *StABL1* transgenic plants. Data collected at 5 and 10 DAT to SDs. *n* = 6. The error bar indicates the standard deviation. E, Percentage of soil-grown WT E3 and *StABL1* transgenic plants with tubers. Data were obtained at 5 and 10 DAT to SD after 4 weeks grown under LD conditions., *n* = 6. Three independent experiments were performed. F, Representative photos of *StABL1*-overexpressing plants and WT E3 plants grown in pots under LDs for 50 and 90 d in net house. Scale bar: 25 cm. G, Percentage of plant survival with the plant aging (DAP) in WT E3 and *StABL1ox* transgenic plants, *n* = 24. H, Representative photos of *StABL1ox* plants and WT control E109 plants grown in pots for 40 d under LDs. Scale bar: 10 cm. I, Percentage of plant with flower buds in *StABL1ox* plants and its WT control E109, *n* = 8. J, Percentage of plant with open flower in *StABL1ox* plants and its WT control E109, *n* = 8. The asterisks in (B and D) indicate a statistically significant difference (Student’s *t* test, *P < 0.05, **P < 0.01).

**Figure 3:**
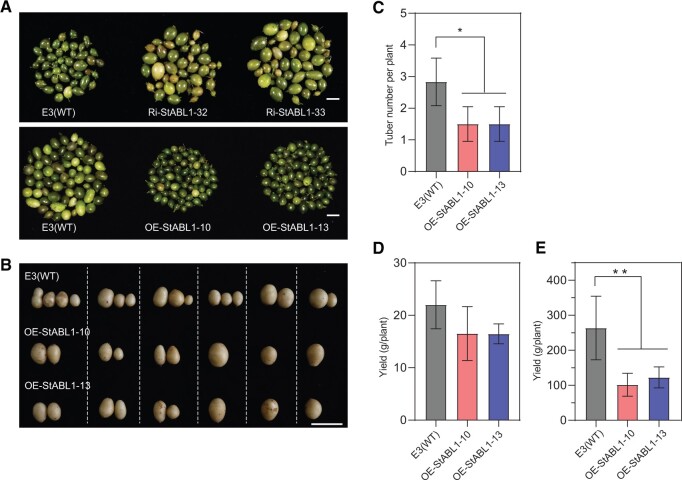
Tuber phenotype of WT and *StABL1* transformants. A, Representative microtuber photographs of WT E3 and *StABL1* transgenic plants, grown in 8% sucrose MS medium for 70 d under SD conditions. Scale bar: 1 cm. B, Representative tuber photographs of six soil-grown WT E3 and *StABL1-*overexpressing transgenic plants. Picture was taken at 60 DAT to SDs. C, Tuber number per plant and (D) yield for soil-grown WT E3 and *StABL1-*overexpressing transgenic plants. Data were obtained at 60 DAT to SDs with six plants for each genotype. Data are presented as mean ± standard deviation. E, Yield for net house-grown WT E3 and *StABL1*-overexpressing transgenic plants. Data were obtained at 120 DAP, *n* ≥ 14. Data are presented as mean ± standard deviation. The asterisks indicate a statistically significant difference (Student’s *t* test, *P < 0.05, **P < 0.01).

Then, the transgenic lines were investigated for tuberization in vitro under SD conditions. The tuberization time of these OE plants was significantly accelerated compared with that of WT plants (Figure 2, A and B). Although the microtuber size or single microtuber weight was increased in *StABL1* RNAi plants and decreased in OE plants, there was no significant variation in tuberization time between *StABL1* RNAi plants and WT E3 (Figures 2, A, B and 3, A). Furthermore, we found that these transgenic lines (both OE and RNAi plants) and the untransformed WT E3 did not form microtubers in vitro under LD conditions. Second, the initiation of tuber formation occurred much earlier in OE plants than in WT E3 plants in vivo after transferred to SD conditions, and more swollen tubers were observed in OE plants at the initiation stage (Figure 2, C–E) as a result of more axillary tuber formation along stolons (Supplemental Figure S4A). These results suggest that overexpression of *StABL1* in potato can strongly promote tuber initiation. Interestingly, the onset of leaf senescence was also much earlier, and the total length of the plant life cycle was substantially shorter than that of the WT E3 grown in net house under natural LD conditions (Figure 2, F and G; Supplemental Figure S4E), indicating an early maturity phenotype in *StABL1*-overexpressing plants. In parallel, early maturation led to a lower tuber yield than that of the WT E3 plants (Figure 3, B–E).

To explore the potential role of *StABL1* in photoperiodic tuberization, we generated GFP-fused *StABL1*-overexpressing transgenic lines in the E109 background (Supplemental Figure S3B), a strict SD genotype that only form tubers under SDs (Zhou et al., 2019). However, no tubers formed in the transgenic lines and untransformed potato cultivar E109 plants under LDs both in vitro and in vivo. Interestingly, the flowering transition was substantially promoted in the OE plants compared with WT E109 under LD conditions (Figure 2, H–J). Thus, more open flowers were found in OE plants at the observed time (Supplemental Figure S4, B and C). In addition, the floral transition was also promoted in *StABL1*-overexpressing transgenic lines relative to WT E109 under SDs (Supplemental Figure S4D). Therefore, we speculated that StABL1 might interact with florigen StSP3D, which is involved in day-neutral flowering in potato (Navarro et al., 2011). To examine this hypothesis, we used Y2H and BiFC assays to test their interaction and found that StABL1 and StSP3D can interact with each other (Figure 1, D–F). Collectively, these results indicate that overexpression of *StABL1* can strongly promote early tuberization and floral transition, and consequently early maturity in potato.

### Genome-wide identification of StABL1 binding sites

**Figure 4:**
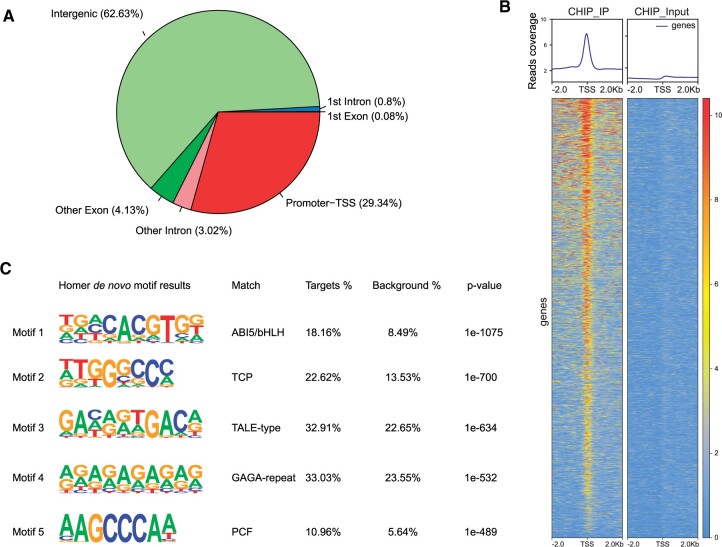
Genome-wide identification of StABL1 binding sites. A, Distribution of peak in functional regions of genome. Promoter-TSS: ±2,000 bp of TSS; TES: ±1,000 bp of TES; Intergenic: gene-free region from 1,000-bp downstream of TES to 2,000-bp upstream of the TSS of the closest gene. Exon and intron are corresponding to the gene models in genome annotation. B, Heat maps showing the ChIP-Seq distribution of reads across TSS. C, HOMER de novo motif enrichment analyses of StABL1 binding peaks. The top five significantly enriched binding motifs and their matched TF family were presented.

Because the genetic regulatory networks that underlie tuberization in potato have been described clearer than that of flowering (Zierer et al., 2021), StABL1-mediated tuberization transition was focused in this study. To investigate the molecular mechanism underlying the functions of *StABL1* in tuberization onset, we performed chromatin immunoprecipitation sequencing (ChIP-Seq) analysis using a *35S: GFP-StABL1* transgenic line (OE-StABL1–13) to identify the binding sites of StABL1. Under induction conditions, 3-week-old in vitro plants shortly before tuberization were sampled for library preparation and sequencing. A total of ∼30.9 and ∼31.1 million clean reads were produced from immunoprecipitation (IP) and input libraries, respectively. Approximately 21 million (70.2%) and 19 M (60.6%) reads were mapped to unique positions in the potato DM genome (Genome assembly DM version 6.01), with most of the reads distributed in intergenic regions (Supplemental Figure S5A). The MACS2 peak calling tool revealed 53,037 peaks located on all chromosomes, with an increased density of peaks trending toward the distal regions of the chromosomes (Supplemental Figure S5B). The genomic locations of the peaks revealed that 29.34% of the peaks were close to the transcription start site (TSS; Figure 4A), while a large proportion (62.63%) of the peaks were located in intergenic regions. To investigate the detailed StABL1 binding profile in the promoter region, read distribution and peak profile analyses revealed that StABL1 binding sites were mostly located toward the TSS (Figure 4B). To identify the StABL1-binding motifs, we used HOMER (Heinz et al., 2010), which scores a list of motifs within ChIP-Seq peaks by computing the enrichment of motifs with background sequences, to characterize the known or de novo sequence motifs. The top five motifs from the HOMER known motif enrichment analysis showed that the bZIP/bHLH and TCP TF-binding motifs were strongly enriched (Supplemental Figure 5C). Consistently, the de novo sequence motif analysis showed similar results: the ABI5/bHLH, TCP, and TALE-type TF-binding motifs were overrepresented (Figure 4C). The binding of StABL1 mainly to the core CACGTG motif was consistent with our previous finding (Liu et al., 2019), in which StABL1 directly binds to the G-box motif, as confirmed by electrophoretic mobility shift assays (EMSAs).

### Identification of tuberization-related genes and pathways regulated by StABL1

**Figure 5:**
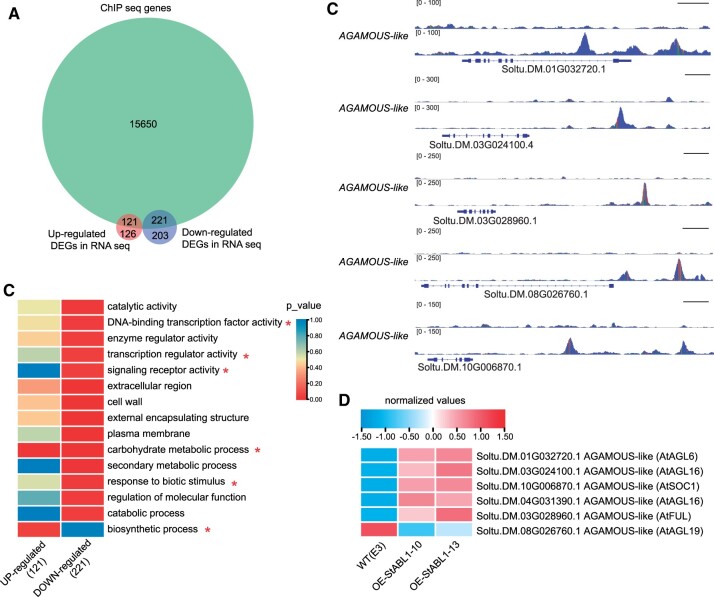
Identification of StABL1 target genes. A, Venn diagram showing the number and overlapping genes between the StABL1-binding targets revealed by ChIP-Seq and the DEGs, identified by RNA-Seq in *StABL1*-overexpressing lines. B, Heatmap showing the *P*-values of the functional categories significantly enriched (*P* < 0.05) in either upregulated or downregulated DEGs in StABL1-overexpressing lines. *P*-values were calculated by one-side Fisher’s exact test. C, StABL1 binding profiles to *AGAMOUS-*like genes visualized with the IGV. Scale bar: 1 kb. D, Heatmap showing the normalized expression levels of *AGAMOUS-*like genes. Genes are represented with their gene ID, annotation, and Arabidopsis homolog. Data represent the mean of three CPM values in RNA-Seq.

To identify StABL1 target genes based on ChIP-Seq data, genes that contain one or more peaks within 2-kb upstream of the TSS to 1-kb downstream of the transcription end site (TES) region are referred to as StABL1-targeted genes. In total, we identified 15,992 StABL1-targeted genes (Supplemental Data set 1). To further identify genes regulated by StABL1 in tuberization, WT E3 and two *StABL1*-overexpressing transgenic plants sampled at the same time as the ChIP assays were used for RNA-Seq. By pairwise comparisons of the RNA-Seq data, a total of 247 upregulated and 424 downregulated genes were identified in *StABL1*-overexpressing plants (Supplemental Data set 2). Further comparing the target genes from the ChIP-Seq data and differentially expressed genes (DEGs) from RNA-Seq revealed 342 overlapping genes (Figure 5A), of which 121 genes were upregulated and 221 genes were downregulated in *StABL1*-overexpressing plants.

Gene ontology (GO) analysis of these overlapping genes showed that GO terms related to biosynthetic process were enriched in StABL1 upregulated genes, and transcription regulator activity, signaling receptor activity, and response to biotic stress were enriched in StABL1 downregulated genes, while carbohydrate metabolic process was enriched in both StABL1 upregulated and downregulated genes (Figure 5B). These results are consistent with the physiological characteristics of tuber initiation, which are associated with changes in the activity of enzymes involved in sugar metabolism and the accumulation of large amounts of storage compounds. The enriched GO terms for the genes involved in signaling receptor activity included ABA receptor *PYR1* genes that were targeted and downregulated by StABL1 (Supplemental Figure S6, A and B), indicating a negative feedback regulation by StABL1 to control the accumulation of specific *PYR* receptors. In addition, we observed that several differentially expressed *AGAMOUS-like* genes were enriched in TF activity GO terms and were targeted by StABL1 (Figure 5, C and D). Their homologs in Arabidopsis are *SOC1*, *FUL*, and *AGAMOUS-like*, which are proposed as downstream targets of the FLOWERING LOCUS T-FLOWERING LOCUS D (FT-FD) FAC during the floral transition (Teper-Bamnolker and Samach, 2005; Andres and Coupland, 2012). The expression of *FUL* was further confirmed by RT-quantitative polymerase chain reaction (RT-qPCR; Supplemental Figure S6C). Taken together, these results suggest that biosynthetic processes, phytohormone responses, and flowering pathways were modulated by StABL1.

### StABL1 activates *StGA2ox1* and modulates gibberellin metabolism in potato

**Figure 6:**
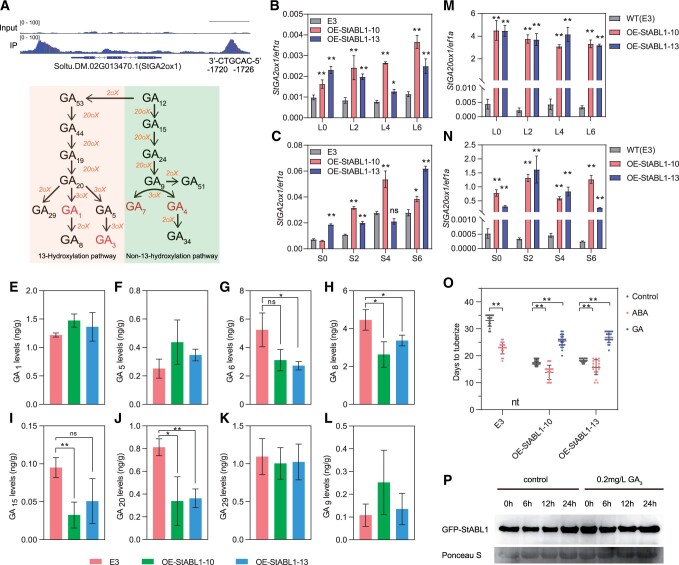
Gibberellin metabolism and activity is affected in *StABL1*-overexpressing plants. A, StABL1 binding profile and control (input) peaks at *StGA2ox1* visualized with the IGV. The position of G-box motif was indicated from the TSS. Scale bar: 1 kb. B and C, Time course of *StGA2ox1* relative expression in WT E3 and *StABL1*-overexpressing plant leaves (B) and stolon tips (C). L0, L2, L4, and L6 represent leaves sampled at 0, 2, 4, and 6 DAT to SD, respectively. S0, S2, S4, and S6 represent stolon tips sampled at 0, 2, 4, and 6 DAT to SD, respectively. Data are presented as mean ± standard deviation, *n* = 3. D, Major reactions in GA biosynthetic pathway (Yamaguchi, 2008). E–L, The endogenous levels of gibberellins in WT E3 and *StABL1*-overexpressing stolon tips which were sampled at 7 DAT to SD. Data are presented as mean ± standard deviation, *n* = 3. M and N, Time course of *StGA20ox1* relative expression in WT E3 and *StABL1*-overexpressing plant leaves (M) and stolon tips (N) sample mentioned in (B and C). O, Microtuber initiation time of WT E3 and *StABL1*-overexpressing plants on tuber-inducing medium (Control) or tuber-inducing medium supplemented with 0.2 mg L^−1^ GA_3_, or 5-μM ABA. Data are presented as mean ± standard deviation, *n* ≥ 30. nt represents no tuber. All the asterisks in this figure indicate a statistically significant difference (Student’s *t* test, *P < 0.05, **P < 0.01). ns means no significant difference. P, Western blot detection of GFP-StABL1 protein in lowest node of OE-StABL1–13 line under control and GA_3_ treatment within 24 h. Rubisco large subunit (Rbc L) was used as a loading control.

GA is the best recognized phytohormone that represses tuberization. Tuber marker gene *StGA2ox1* is involved in tuberization time and promoted by long-distance signal StSP6A in stolon tips (Kloosterman et al., 2007; Navarro et al., 2011)*.* To explore the potential role of *StABL1* in gibberellin responses, first, we observed StABL1 binding peak substantially enriched in the promoter of *StGA2ox1* based on ChIP-Seq data (Figure 6A). To confirm whether the expression of *StGA2ox1* is regulated by StABL1, the time course of *StGA2ox1* relative expression in WT E3 and *StABL1-*overexpressing plant was analyzed. The results showed that *StGA2ox1* transcripts were induced largely in stolon tips of E3 plants under SDs. In addition, transcripts for *StSP6A* were induced quickly in leaves, and slightly delayed in stolon tips with respect to the leaves (Supplemental Figure S7). Moreover, the expression of *StGA2ox1* was upregulated in *StABL1* OE lines in both leaves and stolon tips in relative to E3 (Figure 6, B and C), while the expression of *StGA2ox1* in leaves was much lower. These results indicate that StABL1 may regulate *StGA2ox1* expression directly.

As GA2-oxidase catalyzes the hydroxylation of the C-2 of active C19-GAs, including GA_1_ and GA_4_ and their immediate precursors GA_20_ and GA_9_ (Figure 6D), to produce biologically inactive GAs (Yamaguchi, 2008), we speculated that gibberellin homeostasis is regulated by the SP6A-StABL1 molecular module in potato. To demonstrate the role of *StABL1* in affecting endogenous GAs, we profiled GA content in the stolons of *StABL1*-overexpressing lines and E3 at 7 d after transferred (DAT) to SDs. Eight of 16 measured GAs were absent or only present in trace amounts. GA_6_, GA_8_, GA_15_, and GA_20_ were decreased in the transgenic lines, while no significant variations in GA_1_, GA_5_, GA_9_, and GA_29_ were found between the transgenic lines and WT E3 (Figure 6, E–L). These results completely matched the GA profile of *StGA2ox1-*overexpressing lines, as previously reported (Kloosterman et al., 2007). In addition, we found that the expression of *StGA20ox1* was dramatically upregulated (Figure 6, M and N). This gene is under negative-feedback control by the biosynthetic end-product active GAs (Carrera et al., 1999). Moreover, whether the *StABL1*-overexpressing lines were more resistant to active GA treatment than WT E3 with respect to tuberization was also tested. In the medium supplemented with 0.2 mg L^−1^ GA_3_, no tubers formed in WT. But the tuberization time in *StABL1* transgenic plants with GA treatment was almost recovered to that of WT under control condition (Figure 6O). To check the possibility that GA treatment might reduce StABL1 protein abundance, we used GFP-tagged StABL1 OE transgenic lines and found no obvious variation in StABL1 protein levels between the control and GA treatments within 24 h (Figure 6P). Taken together, these results suggest that GA activity was largely blocked in *StABL1* transgenic plants.

## Discussion

### StABL1, an FD closely related AREB/ABF/ABI5 subgroup of group A bZIP TFs, forms a complex with FT-like proteins

The photoperiodic-mediated systemic signal FT regulates multiple developmental transitions, such as flowering time (Kardailsky et al., 1999), growth cessation in trees (Bohlenius et al., 2006), meristem termination (Shalit et al., 2009), and storage organ formation (Navarro et al., 2011), primarily by interacting with FD via the 14-3-3 protein. In addition, some other bZIP TFs have already been described to form aFACs with the florigen to control flowering or other developmental processes (Park et al., 2014; Li et al., 2015; Tylewicz et al., 2015; Brambilla et al., 2017). In this study, we characterized StABL1 as a component to form aTAC and aFAC. The interaction mode of StABL1 with StSP6A and StSP3D is identical to that of OsFD in rice (Taoka et al., 2011), but different from that of StFDL1, which interacts with St14a mainly in the cytoplasm (Teo et al., 2017). A recent study reveals that FT can promote flowering independently of FD and FDP, and possibly act through other members of the bZIP family (Romera-Branchat et al., 2020). In addition, an *StFDL1*-independent pathway in tuberization is proposed (Teo et al., 2017). These studies and our findings strongly indicate that group A bZIP TFs contribute to the formation of alternative FACs or TACs. Interestingly, StCEN was also observed to physically interact with StABL1, but StFD or StFDL1 could not interact with StABL1 in the Y2H interaction (Supplemental Figure S8). Because StCEN suppresses both flowering and tuberization and binds components of TAC (Zhang et al., 2020). It is likely that StCEN also act as inhibitors of the aTAC/aFAC complexes.

Two independent ChIP-Seq data for AtFD show that the core G-box motif CACGTG is enriched (Collani et al., 2019; Romera-Branchat et al., 2020), which is similar to G-box related ABA-responsive elements, recognized by AREB/ABF/ABI5 members (Song et al., 2016). In this study, a core G-box CACGTG at the center of the represented motif, analyzed by ChIP-Seq reads (Figure 4C), was the most enriched binding site for StABL1. The binding of StABL1 to the core G-box CACGTG motif is confirmed in our previous study by EMSA (Liu et al., 2019). Remarkably, the TCP and TALE-type TF-binding motifs were also enriched in StABL1 ChIP-Seq peaks (Figure 4C), possibly due to the combinatorial binding of StABL1 to these TFs. In fact, the TCP TF StBRC1b and TALE TF StBEL5 are involved in tuberization control (Chen et al., 2003; Michael et al., 2021). Genetic and molecular analyses indicate that class II CIN TCP TFs function synergistically with FT and FD to positively regulate flowering in Arabidopsis (Li et al., 2019). Therefore, the potential interaction of the FT-StABL1 complex with StBRC1b or other TCP TFs, and their contribution to the separate effects on tuberization and plant lifecycle deserve further investigation.

### StABL1 links the two core hormone systems: florigen and GA

Potato tuber formation is associated with sucrose unloading switches, meristem growth cessation, and changes in phytohormone content. The interaction of StSP6A with StSWEET11 and StFDL1 provides insights into molecular mechanism for StSP6A action (Zierer et al., 2021). However, the mechanism of hormone-level regulation, which is critical for tuber formation, remains largely unknown. ABA is reported as a tuberization stimulator but not a tuberization inducer, by counteracting the inhibitory effect of GA (Aksenova et al., 2012). GA inhibits tuberization by constraining cortical microtubule reorientation at the sub-apical region of stolon (Fujino et al., 1995; Sanz et al., 1996). *StGA2ox1*, induced locally in stolons by StSP6A, is promoted shortly before stolon swelling (Kloosterman et al., 2007). In our results, *StGA2ox1* was upregulated in *StABL1*-overexpressing plants in both leaves and stolons. The gibberellin profile of *StABL1*-overexpressing plants perfectly matched that of *StGA2ox1*-overexpressing plants. Despite a lack of evidence that direct activation of *StGA2ox1* by StABL1, as a results of almost no promoter activity of *StGA2ox1* in *N. benthamiana* leaves based on our dual luciferase reporter assay.

Our findings suggest that *StGA2ox1* functions downstream of the StSP6A–StABL1 complex to promote tuberization. This hypothesis is also supported by the fact that *StABL1*-overexpressing plants were hyposensitive to GA, as evidenced by tuber formed in *StABL1*-overexpressing plants under GA treatment; the largely upregulated *StGA20ox1*, which is negative-feedback regulated by GA activity in potato; And the physiological responses to SD were attenuated in *StABL1*-overexpressing plants (Supplemental Figure S9, A–J). Thus, the association of StABL1 with StSP6A suggests that ABA can function in the photoperiodic pathway to plastically tune tuber development, although to what extent the function of StABL1 depends on its interaction with StSP6A and StSP3D remains elusive, which can be realized by expressing *StABL1* in *sp6a or sp3d* null mutant backgrounds. However, there are still many experimental difficulties to do gene editing in heterozygous and self-incompatible tetraploid potato genome at present.

It has been proposed that ABA-regulated actions but not flowering induction represent ancestral PEBP functions for sexual reproduction, which evolves relatively late in plant evolution (Khosa et al., 2021). In fact, PEBP members are reported to regulate seed germination by affecting ABA and GA responses (Xi et al., 2010; Chen et al., 2021), and FD or FD-like proteins are involved in the ABA response in Arabidopsis or hybrid aspen trees (Tylewicz et al., 2015; Romera-Branchat et al., 2020), supporting an ancient role of FT- and FT-interacting bZIP TFs in ABA responses. Thus, the FT interaction with AREB/ABF/ABI5 family members might be a conserved molecular mode.

### The interaction of StABL1 and FT-like contributes to synchronize potato maturity syndrome

Flowering transition, the onset of tuberization, leaf senescence, and life cycle length were regulated by StABL1. Overexpression of *StABL1* in E3 promoted early tuberization under SDs and showed a short life cycle length; and promoted early flowering in the strict SD potato genotype E109. While the flowering time exhibited no substantial variation in the transgenic lines of the E3 background, likely due to the *StSP3D*-controlled flowering pathway is compromised in E3 plants. The *StABL1* RNAi transgenic plants made larger microtubers but tuberized at the same time as WT E3 plants, which might be attributed to the residual expression of *StABL1*, or genetic redundancy between *StABL1*, *StFDL1*, and genes encoding closely related group A bZIP TFs. In addition, although these *StABL1-*overexpressing plants in SD genotype E109 background flowered earlier compared with WT under natural LDs, we did not observe an obvious effect on life cycle length (Supplemental Figure S4F). Mostly, the transition to flowering dictates a global redistribution of resources, signals to create reproductive organs. Given that the potato genotypes used in this study are self-incompatible tetraploid potatoes. So we hypothesize that the life cycle length should be shorter under LDs when expressing *StABL1* in self-compatible SD potatoes. Here, we describe that *StABL1*-overexpressing transgenic plants showed accelerated signs of senescence, and completed their life cycle much earlier than WT plants (Figure 2F). Posttuberization senescence is thought to be a normal physiological response in potato (Kloosterman et al., 2013). Additionally, we found the *SOC1* and *FUL* genes were upregulated in *StABL1*-overexpressing plants (Figure 5D). These genes have been found to mediate meristem determinacy and annual growth habits by inhibiting longevity in addition to flowering time (Melzer et al., 2008; Balanza et al., 2018). Thus, StABL1-mediated *FUL* and *SOC1* expression could be one of the mechanisms of aFAC/aTAC to synchronize potato maturity syndrome, a hypothesis that need to test in future work.

**Figure 7:**
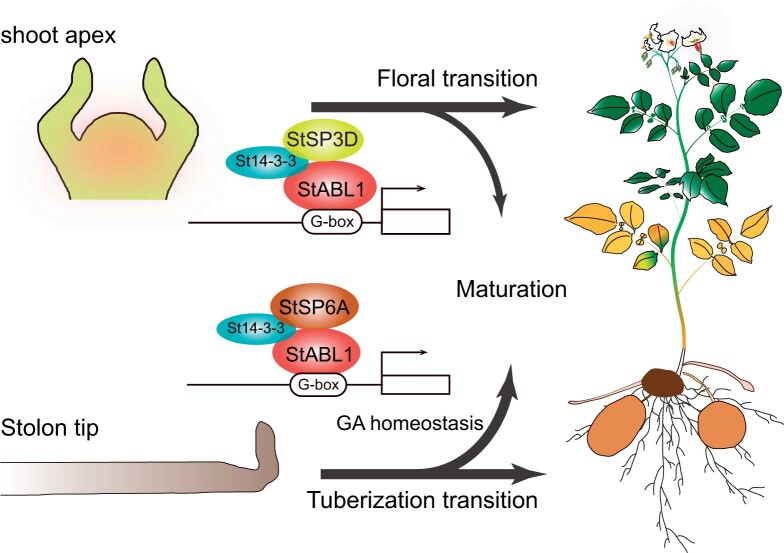
Model for aTAC and aFAC in promoting potato maturation. Under SD conditions, the accumulated StSP6A interact with StABL1 bridged by St14-3-3s, to form aTAC, which block GA activity in stolon tips by altering GA metabolism, to promote tuberization transition; under both LD and SD conditions, the expressed StSP3D associate with StABL1 to trigger floral transition. Collectively, StABL1 functions with florigen (StSP3D) and tuberigen (StSP6A) to promote flowering and tuberization and thereby synchronizes potato maturity syndrome in potato.

In summary, we identified StABL1 as a signaling integrator of *FT-*like genes in potato to form aTAC or aFAC, which is involved in flowering, tuberization, and maturity control by modulating ABA and GA responses. Our findings suggest that StABL1 associates with StSP6A and StSP3D to synchronize the potato maturity syndrome, by promoting dual reproduction in potato (Figure 7). Thus, exploring the natural variants or creating elite alleles of *StABL1* using gene editing will be a viable approach for breeding potato varieties for different geographic regions and harvest times.

## Materials and methods

### Plant materials and growth conditions

Two previously described potato (*S.* *tuberosum* L.) varieties (Zhou et al., 2019) were used in the present research: E109sm (E109) and E-potato 3 (E3). Plants were propagated in vitro using single-node stem on MS medium supplemented with 3% (w/v) sucrose under a photoperiod of 16 h of light/8 h of dark at 20°C, with light intensities ranging from 400 to 1,000 μmol m^−2^ s^−1^. Each experiment was designed with either three independent replicates of six plants (pot-grown plants for in vivo tests) or at least 72 plantlets (nine plantlets in each culture box for in vitro tests).

For the in vitro tuberization assay, single nodes were cultured on tuber-inducing medium: 8% (m/v) sucrose MS medium supplemented with 0.2% active carbon. Tuberized plants were recorded weekly starting from the fourth week. Microtubers were harvested 10 weeks after propagation (WAP). For phytohormone treatment, plants were propagated in vitro using single node on MS liquid medium with 3% sucrose using PCR plates with cut tube tips. After growth under LD conditions for 2 weeks, plants were transferred to MS liquid medium with 8% sucrose supplemented or not with phytohormones for tuber induction under SD conditions. Tuberized plants were recorded every day after treatment.

For the in vivo tuberization tests, 3-week-old WT and transgenic plantlets were transplanted into plastic pots with a diameter of 10 cm (one plant per pot) in one tray (8 pots for each genotype) in a growth room with an LD photoperiod (16-h/8-h light/dark) at 20 ± 2°C and were managed to ensure normal growth. Then, plants at the 10-leaf stage (∼4 weeks) were transferred to an SD photoperiod (8-h/16-h light/dark) at 20 ± 2°C. Tuberized plants were recorded every 5 d. For time-course gene expression experiments, the fifth expand leaf with two leaflets from the shoot apex and the apex of the stolon from plants grown in the growth room were sampled at 2 h after light on. The samples were immediately frozen in liquid nitrogen and stored at −70°C until use. Tubers were harvested 10 weeks after transplantation. For plants grown in a net house, sprouting tubers were planted in pots with a diameter of 25 cm (one plant per pot) and were managed to ensure normal growth.

### Quantification of plant maturity, internode length, and tuber yield

For plant maturity, plant survival in relation to the number of days after planting (DAP) was measured every 3–4 d. For plant height, from the soil surface to the tip of the plant was measured. For internode length, the first expanded leaf from the shoot apex was counted as the first internode. For tuber yield, tuber fresh weight per plant was quantified for at least 6 plants in a growth room and 14 plants in a net house after growth for 12 weeks.

### Vector construction and potato transformation

All genes of interest were amplified by PCR using cDNA from E3, except for *StSP3D*, which was cloned from E109 plants as a template. PCRs were performed with Phanta Super-Fidelity DNA Polymerase (Vazyme, Nanjing, China) according to the manufacturer’s instructions. The primers used for amplification are shown in (Supplemental Table S1). To generate *StABL1*-*Ri* transgenic plants, double-stranded RNA interference was used to silence the *StABL1* genes. To construct the RNA interference vector, the corresponding fragments amplified from complementary DNA (161 to 561 bp) were cloned into the pHellsgate8 vector and digested with XbaI and XhoI using ExnaseII (Vazyme) according to the manufacturer’s recommendations. The vector was introduced into *Agrobacterium tumefaciens* strain GV3101 and transformed into E3. To create *35S* promoter-driven *StABL1*-overexpressing transgenic plants, the coding sequence of *StABL1* was amplified from complementary DNA of E3 fused with GFP at the N-terminus (*35S: GFP-StABL1*) through overlapping PCR and inserted into pBI121 that was digested with BamHI and SacI via a restriction–ligation method. The construct was introduced into *A.* *tumefaciens* strain GV3101, which was then transformed into the E109 and E3 lines, as previously described (Si et al., 2003).

### Y2H assays

The full-length or truncated coding sequence of *StSP6A*, mutated *StSP6A, StSP3D*, and *StABL1* was cloned into the EcoRI and SalI sites of the pGBKT7 vector, and site-directed mutagenesis was performed with the primers listed in (Supplemental Table S1). The full-length coding sequences of *St14a*, *St14f*, *StSP3D*, *StFD*, *StFDL*, and *StCEN* were cloned into the EcoRI and BamHI sites of the pGADT7 vector. These pairwise combinations or the corresponding empty vectors were cotransformed into the AH109 yeast strain using the BD Matchmaker Screening Kit according to the manufacturer’s protocols.

### BiFC

The open reading frames of *StSP6A*, mutated *StSP6A*, *StSP3D*, *St14a*, and *StABL1* were amplified using specific primers (Supplemental Table S1) and then separately cloned into the NYFP and CYFP vectors via restriction digestion with BamHI and SalI. BiFC vectors were purified by using the Plasmid Miniprep Kit (Zomanbio, www.zomanbio.com). Protoplast isolation from potato leaves and protoplast transformations were performed as described (He et al., 2007) with some modifications. Briefly, the young leaves of 4-week-old E3 plants were cut into strips ∼0.5- to 1.0-mm wide with a fresh sharp razor blade. The prepared enzyme solution (1.5% cellulase R10 (Yakult Honsha, Tokyo, Japan); 0.3% macerozyme R10 (Yakult Honsha); 20-mM KCl (Sigma, St Louis, MO, USA); 20-mM 2-(N-morpholino) ethanesulfonic acid (MES; Sigma), pH 5.7; 0.3 M mannitol (Sigma); 10-mM CaCl_2_ (Sigma); and 0.1% BSA (Sigma)) was added to digest the leaves at 50 R/min in a shaker in the dark for 4–5 h at room temperature. The enzyme solution containing protoplasts was filtered into a new 50-mL centrifuge tube with a 300 mesh cell filter. Samples were spun at 100 × g for 6 min to pellet the protoplasts. Thirty milliliters of W5 solution (115 mM NaCl (Sigma); 94-mM CaCl_2_; 3.75-mM KCl; 1.5-mM MES, pH 5.7) was used to resuspend the protoplasts by gentle shaking. After two washes by centrifugation, resuspended protoplasts in the W5 solution were kept on ice for 30 min. The protoplasts were spun at 100*g* for 6 min and resuspended in mannitol magnesium (MMg) solution (0.3-M mannitol; 15-mM MgCl_2_; 4-mM MES, pH 5.7). Five micrograms of NYFP and 5 µg of CYFP plasmid were gently mixed with 200 µL of prepared protoplasts from the last step in a 2-mL EP tube. Then, 40% PEG4000 solution (40% PEG4000 (Sigma); 150-mM mannitol; 100-mM CaCl_2_) was added to the EP tube, mixed well, and incubated at 23°C for 20 min. Samples were diluted with 2 mL W5 solution and gently mixed. The protoplasts were spun at 100*g* for 6 min and washed again with 2-mL W5 solution. Then, the protoplasts were spun again at 100 *g* for 6 min and gently resuspended in 1-mL W5 solution. Fluorescence was observed using laser confocal fluorescence microscopy (Leica TCS-SP8, Germany) after incubation for 24 h in the dark at room temperature, with experimental setup (lasers, 488 nm; intensity, 8%, collection bandwidth, 497–550 nm; gain value, 300).

### Transient expression in *N. benthamiana*

*Agrobacterium* *tumefaciens* strain GV3101 containing expression vectors was grown overnight in Luria-Bertani medium with appropriate antibiotics at 28°C. The bacteria were pelleted and resuspended in infiltration buffer (10-mM MES, 10-mM MgCl_2_, and 200-μM acetosyringone). The optical density (OD_600_) was adjusted to 0.1 for imaging purposes and 0.4 for immunoblots, IP, and activity assays. For coexpression of multiple constructs, agrobacterial suspensions were equally mixed before infiltration into *N. benthamiana* leaves.

### Co-IP

*Nicotiana* *benthamiana* leaves expressing the appropriate constructs were collected at 2 d postinfection. The cells were homogenized in liquid nitrogen and suspended in 500-μL protein extraction buffer (10% (v/v) glycerol; 25-mM Tris–HCl (pH 7.5); 1-mM EDTA; 150-mM NaCl; 10-mM DTT; 0.2% Nonidet P-40; 1-mM phenylmethylsulfonyl fluoride (PMSF); and protease inhibitor tablets (A32955; Thermo Fisher Scientific, Waltham, MA, USA)) followed by extraction on ice for 30 min and centrifugation for 20 min at 12,000 R/min at 4°C. Twenty microliters of prewashed anti-GFP agarose beads (http://www.ktsm-life.com/, KTSM1301) were added to 400-μL protein extracts and incubated for 2 h at 4°C. Then, the beads were washed 4 times with wash buffer (10% (v/v) glycerol; 25-mM Tris–HCl (pH 7.5); 1-mM EDTA; 150-mM NaCl; 1-mM PMSF; protease inhibitor tablets (A32955; Thermo Fisher Scientific). The immunoprecipitates were eluted with 1 × SDS loading buffer. The samples were boiled at 95°C for 10 min before discarding the beads and subsequently stored at −80°C until western blot analysis.

### Western blot

Protein extraction in potato was performed as described previously (Abelenda et al., 2016). The potato protein samples and the immunoprecipitates were separated on a 10% sodium dodecyl-sulfate polyacrylamide gel electrophoresis (SDS–PAGE) gel and transferred to polyvinylidene fluoride (PVDF) membranes. The membranes were blocked in 5% milk in 1 × TBS (150-mM NaCl, 10-mM Tris–HCl, pH 7.4) with 0.05% (v/v) Tween-20 before hybridization with primary antibodies (anti-GFP mAb, MBL, M048-3) or anti-HA-tagged mAb (MBL, M180-3) at 1:3,000 dilutions. The membrane was washed with 1 × TBST before the addition of the secondary antibody at a 1:3,000 dilution (anti-IgG (H + L chain) (mouse) pAb-HRP, MBL). Enhanced chemiluminescence (ECL) detection was performed according to the manufacturer’s recommendations.

### RNA extraction and RT-qPCR

Total RNA was extracted from the frozen samples using a Total RNApure Kit (ZOMANBIO, http://zomanbio.com). First-strand cDNA was synthesized using a 5 × All-in-One RT Master Mix Reverse Transcription Kit (ABM, http://www.abmgood.com). RT-qPCR was performed on a LightCycler 480 II (Roche, Switzerland) with EvaGreen 2 × qPCR Master Mix (ABM, http://www.abmgood.com). The potato *Ef1α* gene was used as a control gene to normalize the expression data (Nicot et al., 2005). Gene expression levels were calculated via the 2^−ΔCq^ method described by Bio-Rad (Hercules, CA, USA; http://www.bio-rad.com/zh-cn/applications-technologies/real-time-pcr experimental-design). All primer sequences for RT-qPCR analysis are described in (Supplemental Table S2).

### Leaf senescence assay

For the leaf senescence assay, nodes from 3-week-old plants were cut into fresh 2% sucrose (m/v) MS medium. WT and transgenic plant nodes were placed in the same 150-mm Petri dishes; the dishes placed in LD conditions were used as controls, and the dishes in the dark (covered with black bags) were used as treatments. Photographs were taken after the plates were incubated in a growth chamber at 20°C for 5 d. At least 20 nodes for each genotype in one dish were used as one biological replicate. Three biological replicates were used per genotype or treatment.

### Stomatal assays

To determine ABA sensitivity, stomatal aperture bioassays were conducted as previously described (Shin et al., 2011). Briefly, detached leaves from 3-week-old in vitro potato plants were floated in stomatal opening solution (15-mM KCl, 10-mM CaCl_2_, and 10-mM MES-KOH, pH 6.15) in a growth chamber (20°C) under light conditions. After 3 h, the buffer was replaced with fresh stomatal opening solution containing 5-μM ABA or mock solution. After another 1 h of incubation, the abaxial epidermal layers of the leaves were observed using bright-field microscopy (AXIO Observer A1; Zeiss, Oberkochen, Germany), and images were captured. For each treatment or control, a total of 100 stomata in 5 leaves per genotype were measured.

### ChIP analysis

ChIP assays were performed on OE-StABL1–13 potato plants grown in vitro prepared at the same time as RNA-Seq by SeqHealth (Wuhan, China). The shoots of plants grown in vitro were cut into small pieces and fixed in 1% formaldehyde for 10 min at room temperature by a vacuum pump at Zeitgeber time ZT = 2 (ZT2), after which 0.125-M glycine was added, and the mixture was incubated for 5 min to terminate the crosslinking reaction. The sample was then collected, frozen in liquid nitrogen, and homogenized by a tissue lyser. The ground powder was treated with cell lysis buffer, and the nucleus was collected by centrifugation at 2,000*g* for 5 min. Then, the nucleus was treated with nucleus lysis buffer and sonicated to fragment chromatin DNA. Here, 10% of the lysis-sonicated chromatin was stored and named “input,” 80% was used in IP reactions with rabbit polyclonal anti-GFP antibody (Ab290; Abcam, Cambridge, UK, https://www.abcam.com) and named “IP,” and 10% was incubated with rabbit IgG (Cell Signaling Technology, Danvers, MA, USA) as a negative control and named “IgG.” The DNA for input and IP was extracted by the phenol–chloroform method. High-throughput DNA sequencing libraries were prepared by using the VAHTS Universal DNA Library Prep Kit for Illumina version3 (Catalog No. ND607; Vazyme). The library products corresponding to 200–500 bp were enriched, quantified, and finally sequenced on a NovaSeq 6000 sequencer (Illumina, San Diego, CA, USA) with the PE150 model.

Raw sequencing data were first filtered by Trimmomatic (version 0.36), low-quality reads were discarded, and reads contaminated with adaptor sequences were trimmed. The clean reads were used for protein binding site analysis. They were mapped to the reference genome of doubled monoploid potato *S. tuberosum* Group Phureja DM 1-3 516 R44 (version 6.1) from http://solanaceae.plantbiology.msu.edu/dm_v6_1_download.shtml using STAR software (version 2.5.3a) with default parameters. RSeQC (version 2.6) was used for read distribution analysis. MACS2 software (version 2.1.1) was used for peak calling. Bedtools (version 2.25.0) was used for peak annotation and peak distribution analysis. The differential binding peaks were identified by a Python script using Fisher’s test. HOMER (version 4.10) was used for motif analysis (Heinz et al., 2010). Integrative Genomics Viewer (IGV) was used to visualize the signals (Robinson et al., 2011).

### RNA-Seq

For RNA-Seq analysis, 3-week-old (E3, OE-StABL1–10, and OE-StABL1–13) plants grown in vitro under tuber induction conditions shortly before tuber initiation were sampled at ZT2 and homogenized for RNA extraction. Three biological replicates were used for each sample. Two micrograms of total RNA were used for stranded RNA sequencing library preparation using the KC-DigitalTM Stranded mRNA Library Prep Kit for Illumina (Catalog No. DR08502, Wuhan Seqhealth Co., Ltd. China) according to the manufacturer’s instruction. The kit eliminates duplication bias in PCR and sequencing steps by using a unique molecular identifier (UMI) of eight random bases to label the preamplified cDNA molecules. The 200- to 500-bp library products were enriched, quantified, and finally sequenced on a HiSeq X 10 sequencer (Illumina).

After raw data cleaning (filtering out the adaptor and low-quality reads) by Trimmomatic (version 0.36; Bolger et al., 2014) and UMI deduplication of clean reads (in-house software; Ma et al., 2018), the deduplicated consensus sequences were cleaned again by fastap version 0.20.0 to filter out reads shorter than 20 nt (Chen et al., 2018) and used for RNA-Seq analysis. The clean reads were mapped to the reference genome of potato *S. tuberosum* Group Phureja DM 1-3 516 R44 (version 6.1) from http://solanaceae.plantbiology.msu.edu/dm_v6_1_download.shtml using HISAT2. Reads and counts per million (CPM) were calculated using featureCounts version 2.0.0 (Liao et al., 2014) per gene. Genes with at least 1 CPM in >1 sample were considered expressed. After filtering out the genes with low expression abundance (CPM ≤ 1), differential gene expression analysis was performed using the R package edgeR version 3.26.8 (Robinson et al., 2010), and the DEGs were identified (FDR ≤ 0.05 and absolute of log2 (fold change with CPM) ≥0.75). GO enrichment analysis (*P*≤ 0.05) of the DEGs and heatmap drawing were implemented by TBtools (version 1.0.98; Chen et al., 2020).

### Determination of endogenous gibberellin acids levels

Three-week-old in vitro plants were transplanted into plastic pots with a diameter of 10 cm (one plant per pot) in a growth room with an LD photoperiod (16-h/8-hh light/dark) at 20 ± 2°C. After growth for 4 weeks, 10-leaf-stage plants were transferred to an SD photoperiod (8-h/16-hh light/dark) at 20 ± 2°C. Seven DAT to SDs, stolon tips (∼2 cm length) from eight plants were sampled as one replicate. Three replicates of each assay were performed. The collected samples were immediately frozen in liquid nitrogen. Plant materials (50-mg fresh weight) were ground into powder in liquid nitrogen and extracted with 500-μL H_2_O/ACN (acetonitrile). Internal standards were added to plant samples before extraction. The supernatants were collected after centrifugation. The residue was re-extracted by repeating the steps described above. To the resulting solution, 10-µL triethylamine and 10-µL 3-bromopropyltrimethylammonium bromide were added. The reaction solution was vortexed, incubated at 90°C for 1 h, evaporated to dryness under a nitrogen gas stream, redissolved in 100-µL H_2_O/ACN, and filtered through a 0.22-μm filter for further liquid chromatograph-mass spectrometer (LC–MS) analysis. Then, GA contents were detected by MetWare (http://www.metware.cn/) based on the AB Sciex QTRAP 6500 LC–MS/MS platform.

### Accession numbers

Sequence data from this article can be obtained from the Spud DB (http://spuddb.uga.edu/) under the following accession numbers: *StABL1* (Soltu.DM.10G025990.1), *StSP6A* (Soltu.DM.05G026370.1), and *StSP3D* (Soltu.DM.03G011110.1).

## Supplemental data

The following materials are available in the online version of this article.

**Supplemental Figure S1.** Phylogenetic tree and sequence analysis of selection of *StABL1* that putatively forms a transcriptional complex with FT-like paralogs.

**Supplemental Figure S2.** Subcellular co-localization, expression profiles, and binding to 14-3-3s analysis of *StABL1*.

**Supplemental Figure S3.** Characterization of potato *StABL1* transgenic lines.

**Supplemental Figure S4.** Tuberization, flowering, and maturity of *StABL1-*overexpressing transformants.

**Supplemental Figure S5.** Analysis StABL1 binding sites in potato genome.

**Supplemental Figure S6.** Expression analysis of *StABL1*-targeted *PYR1-like* and *AGAMOUS-like* genes.

**Supplemental Figure S7.** Expression analysis of *StSP6A* in WT E3 and *StABL1*-overexpressing plants.

**Supplemental Figure S8.** Y2H assays demonstrating the interaction between StABL1 and StCEN, StFD, and StFDL1 proteins.

**Supplemental Figure S9.** Physiological responses to SD in WT E3 and *StABL1*-overexpressing plants.

**Supplemental Table S1.** List of cloning primers used in this study.

**Supplemental Table S2.** List of RT-qPCR primers used in this study.

**Supplemental Data Set 1.** List of StABL1 binding sites identified by ChIP-Seq.

**Supplemental Data Set 2.** List of DEGs identified using RNA-Seq.

## Supplementary Material

kiac098_Supplementary_Data

## Notes

S.J. designed and performed most of the experiments. X.S. contributed to cloning and potato transformation. Z.C. and L.Y. bioinformatics analysis. H.L. helped with BiFC assay. E.W., P.J., J.Q., and S.B. materials preparation and phenotyping. S.J. and B.S. conceived the project, analyzed the results, and wrote the manuscript. B.S. supervised this study.

The author responsible for distribution of materials integral to the findings presented in this article in accordance with the policy described in the Instructions for Authors (https://academic.oup.com/plphys/pages/general-instructions) is Botao Song (songbotao@mail.hzau.edu.cn).
