# Supplementary material for: Transcription factor StABI5-like 1 binding to the FLOWERING LOCUS T homologs promotes early maturity in potato
Source: Plant Physiol. 2022 Mar 8;189(3):1677–93. doi: 10.1093/plphys/kiac098 (PMC9237700; doi:10.1093/plphys/kiac098)
Supplement: kiac098_Supplementary_Data [file kiac098_supplementary_data.zip › Supplemental Data.pdf]

Supplemental Data

Transcription factor StABI5 like 1 binding to FLOWERING LOCUS T homologs promotes early maturity in potato

Shenglin Jing, Xiaomeng Sun, Liu Yu, Zhengnan Cheng, Huimin Liu, Enshuang Wang, Peng Jiang, Jun Qin, Shahnewaz Begum, Botao Song

Supporting information available for this article

Supplemental Figure S1. Phylogenetic tree and sequence analysis of selection of *StABL1* that putatively forms a transcriptional complex with FT-like paralogues.

Supplemental Figure S2. Subcellular co-localization, expression profiles, and binding to 14-3-3s analysis of *StABL1*.

Supplemental Figure S3. Characterization of potato *StABL1* transgenic lines.

Supplemental Figure S4. Tuberization, flowering, and maturity of *StABL1*-overexpressing transformants.

Supplemental Figure S5. Analysis StABL1 binding sites in potato genome.

Supplemental Figure S6. Expression analysis of *StABL1*-targeted *PYR1-like* and *AGAMOUS-like* genes.

Supplemental Figure S7. Expression analysis of *StSP6A* in wild type E3 and *StABL1*-overexpressing plant.

Supplemental Figure S8. Yeast two-hybrid assays demonstrating the interaction between StABL1 and StCEN, StFD and StFDL1 proteins.

Supplemental Figure S9. Physiological responses to short day in wild type E3 and *StABL1*-overexpressing plants.

Supplemental Table S1. List of cloning primers used in this study.

Supplemental Table S2. List of RT-qPCR primers used in this study.

Supplemental Dataset S1. List of StABL1 binding sites identified by ChIP-Seq.

Supplemental Dataset S2. List of differentially expressed genes (DEGs) identified using RNA-Seq.

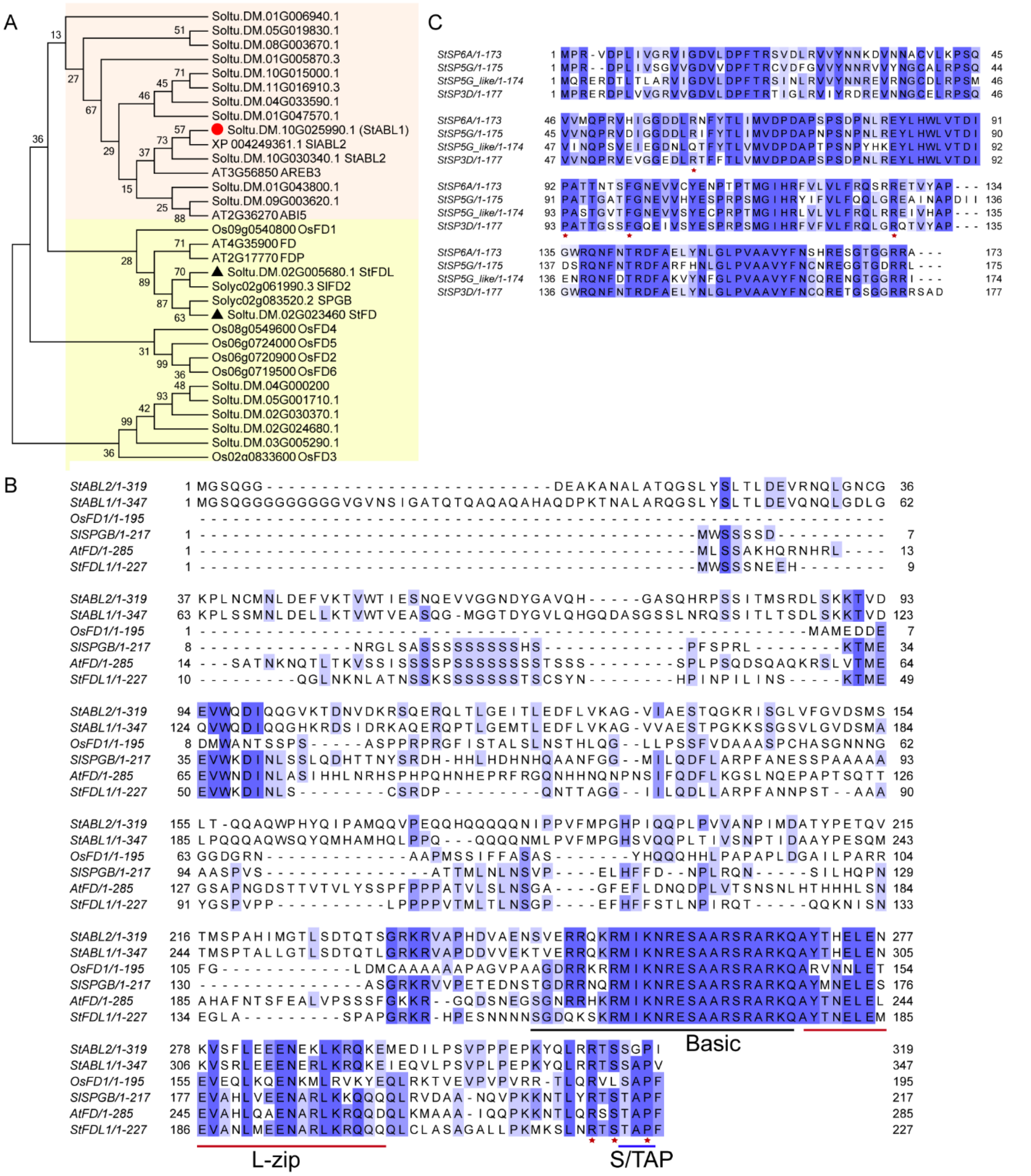

Supplemental Figure S1. Phylogenetic tree and sequence analysis of selection of StABL1 that putatively forms a transcriptional complex with FT-like paralogues. (A) Phylogeny of the *StbZIP*

transcription factors closely related to *StFDL1* and sampled from *Solanum lycopersicum*, *Oryza sativa* and *Arabidopsis thaliana*. MEGA6 with the Neighbor-Joining method was used to construct phylogenetic tree. (B) Sequence alignment of potato StABL1, StABL2, StFDL1, rice OsFD1, tomato SPGB, and Arabidopsis AtFD. Conserved bZIP region is highlighted by black line; S/TAP motif is indicated by red line. Conserved 14-3-3-binding motif (-R-S/T-X-S-X-P) is highlighted in red asterisks. Accession number were shown in (A). (C) Sequence alignment of potato StSP6A, StSP5G, StSP5G_like, and StSP3D. The conserved key amino acid residues for 14-3-3 binding to FT are highlighted in red asterisks.

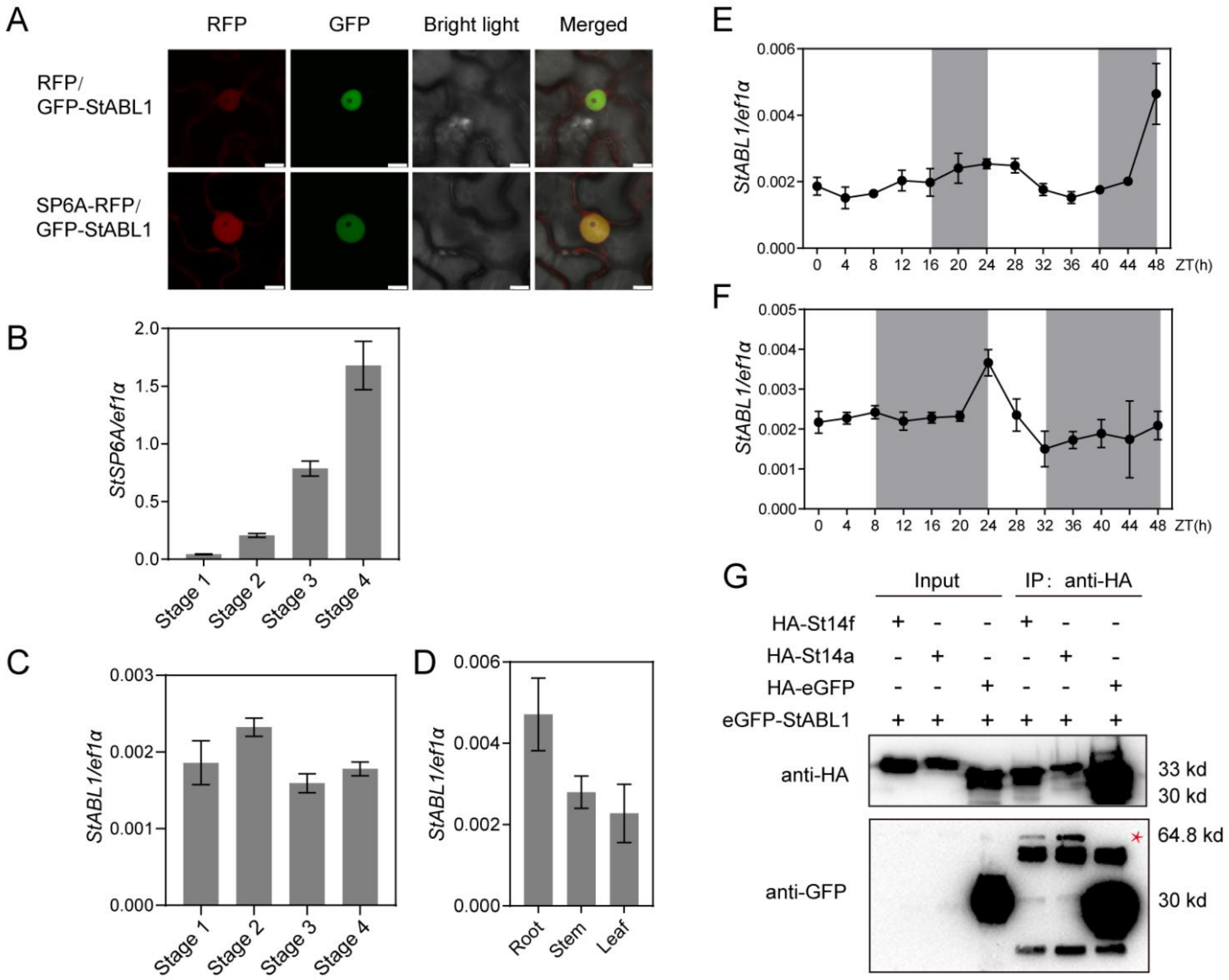

Supplemental Figure S2. Subcellular co-localization, expression profiles, and binding to 14-3-3s analysis of *StABL1*. (A) Subcellular co-localization of the StABL1 protein and StSP6A protein. StSP6A protein was fused to red fluorescent protein (PK7RWG2.0-StSP6A-RFP). StABL1 was fused to green fluorescent protein (PK7WGF2.0-GFP-StABL1), were expressed in *N. benthamiana* leaves. Scale bar: 10 μm. (B-C) Expression of *StSP6A* and *StABL1* in E3 plants grown *in vivo* under short-day conditions (SDs). The samples of different developing stages were collected 7 days after transferred to SDs. Stage 1, stolon with apical hook stage; Stage 2, stolon with open apical hook stage; Stage 3, tuber initiation stage; Stage 4, young tuber stage. Data are presented as mean ± SD. n=3. (D) Expression of *StABL1* in E3 plants grown *in vivo* under SDs. The root, stem and leaf of 4-week-old plants were sampled for RNA extraction. Data are presented as mean ± SD. n=3. (E) Expression of *StABL1* in E3 plantlets grown *in vitro* under long-day conditions (LDs). The whole shoots of 3-week-old plantlets were sampled for RNA

extraction. Data are presented as mean ± SD (standard deviation). n=3. (F) Expression of *StABL1* in E3 plantlets grown *in vitro* under SDs. The whole shoots of 3-week-old plantlets were sampled for RNA extraction. Data are presented as mean ± SD. n=3. (G) Co-immunoprecipitation of StABL1 and St14-3-3s. The proteins were extracted from young coinjected leaves of *N. benthamiana* and immunoprecipitated by anti-HA magnetic beads. Gel blots were probed with anti-HA or anti-GFP antibody. The red asterisk indicates the specific eGFP-StABL1 band. These nonspecific bands represent breakdown products resulting from protein turnover during immunoprecipitation.

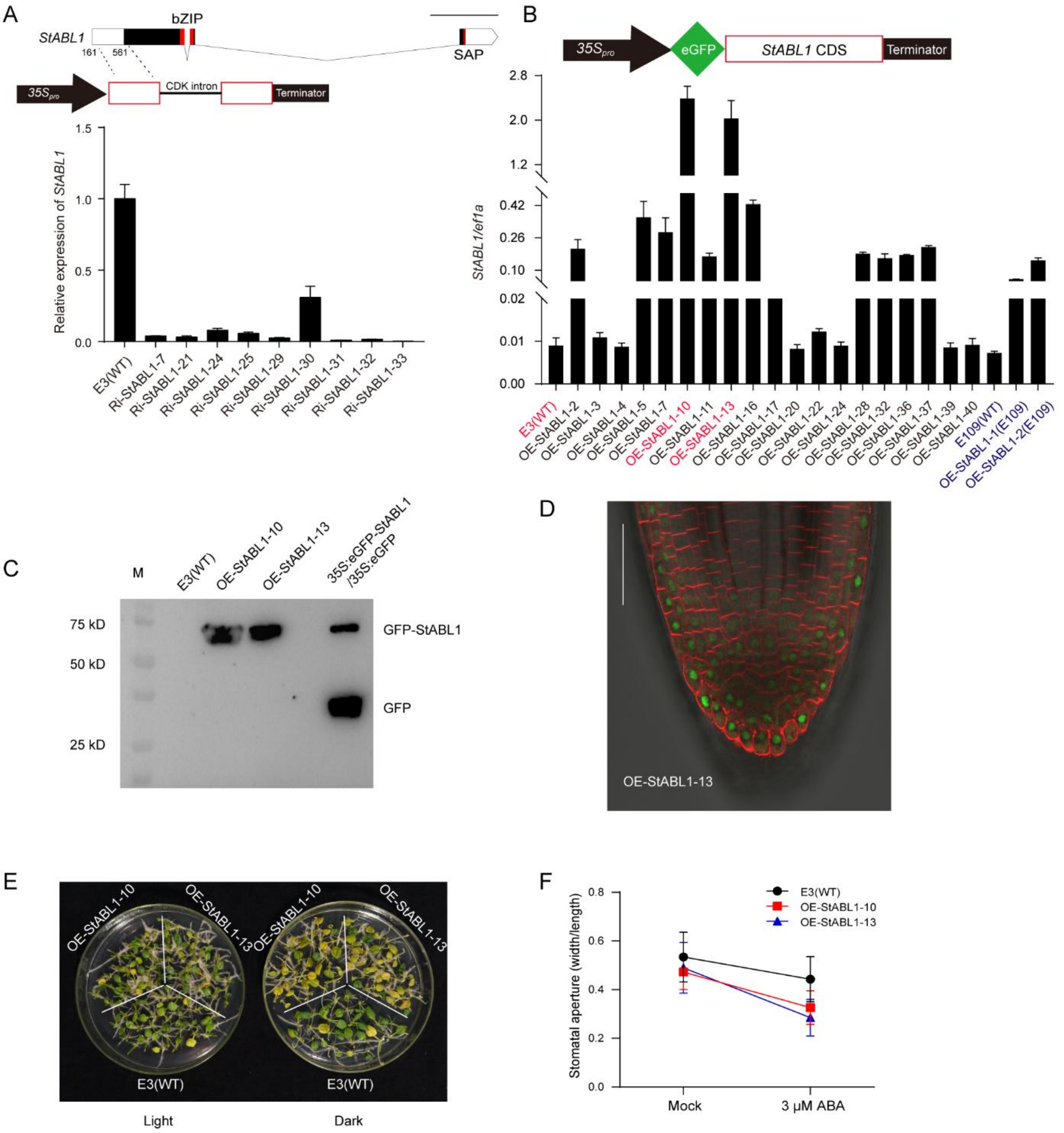

Supplemental Figure S3. Characterization of potato *StABL1* transgenic lines. (A) Gene structure of *StABL1* and assessment of interference efficiency in transgenic lines. The 161 bp to 561 bp of *StABL1* transcript was selected for RNAi vector construct. Scale bar: 1 kb. (B) *StABL1*-overexpressing vector structure and assessment of expression levels in transgenic lines. Transgenic plants picked in E3 background are marked in red; transgenic plants picked in E109 background are marked in blue. (C) Western blot detection of StABL1 protein in wild type E3, overexpression lines. GFP-StABL1 and GFP co-expressed in *N. benthamiana* leaves were

extracted and used as positive control. (D) Root tip of *in vitro* 35*S: GFP-StABL1* potato plant was observed, and imaged using laser confocal fluorescence microscopy (Leica TCS-SPE, Germany) at 488 nm (GFP) or 514 nm (FM 4–64) excitation wavelength after stained with FM4-64 (0.8 μM) for 15 min. Scale bar: 75 μm. (E) Phenotype of the wild type E3 and *StABL1*-overexpressing plant leaves after 5 days of dark treatment and light control, respectively. (F) Quantification analysis for ABA-induced stomatal closure. Stomatal apertures were measured following exposure to 2 μM ABA for 1 h. (≥98 stomatal apertures were measured).

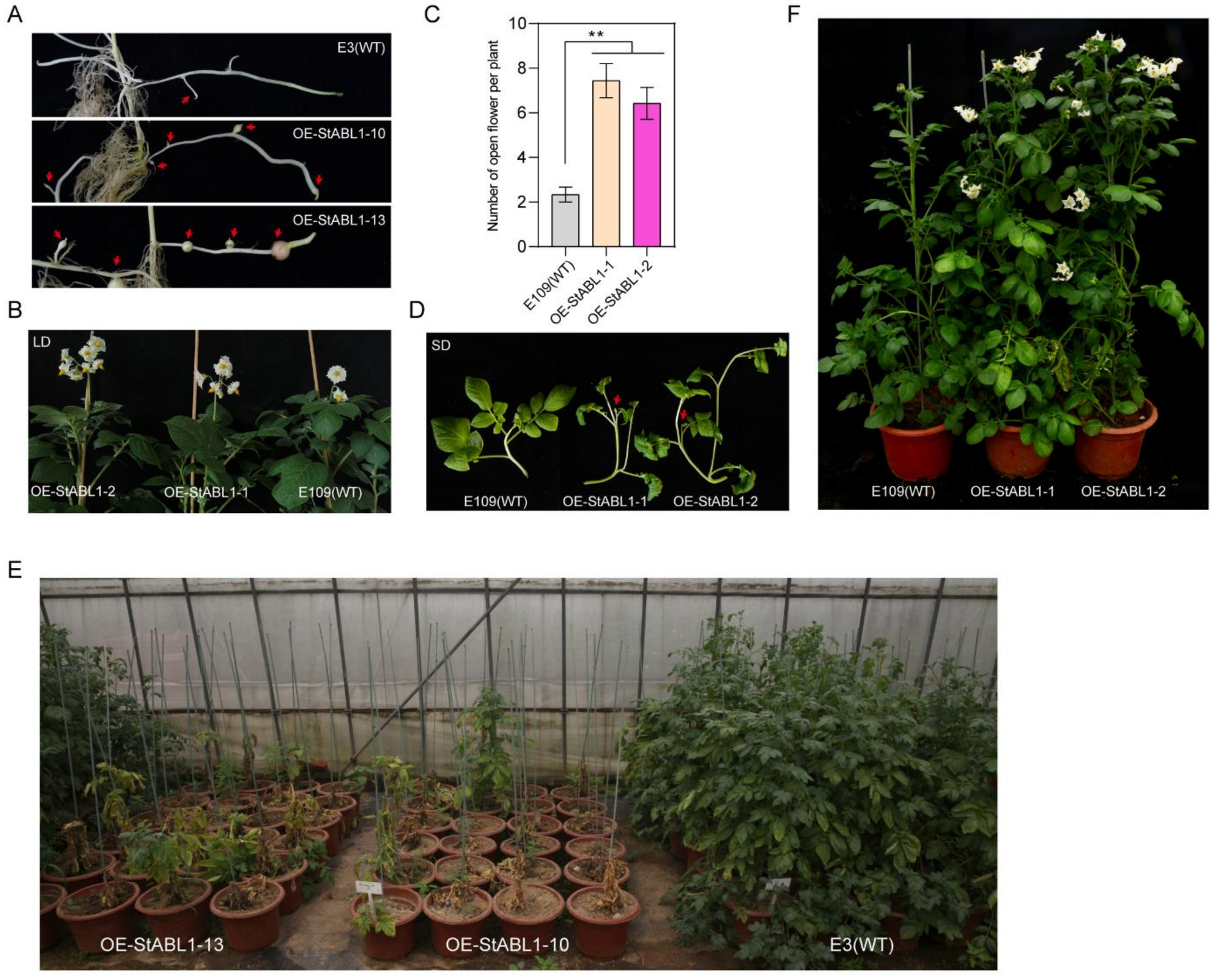

Supplemental Figure S4. Tuberization, flowering, and maturity of *StABL1*-overexpressing transformants. (A) Representative photographs of E3 wild type and *StABL1*-overexpressing transgenic plants at 5 days after transferred (DAT) to short days. (B) Representative flower photographs of E109 wild type and *StABL1*-overexpressing transgenic plants grown under LD (long day) conditions. (C) Average number of open flower per plant. n=8. Data are presented as mean ± SD. The asterisks indicate a statistically significant difference (Student's t-test, $^{**}P < 0.01$). (D) Representative flower photographs of E109 wild type and *StABL1*-overexpressing transgenic plants grown under SD (short day) conditions. (E) Photos of *StABL1*-overexpressing plants and wild type control E3 plants grown in pots under natural LDs for 90 days in net house. (F) Photos of *StABL1*-overexpressing plants and wild type control E109 plants grown in pots under natural LDs for 60 days.

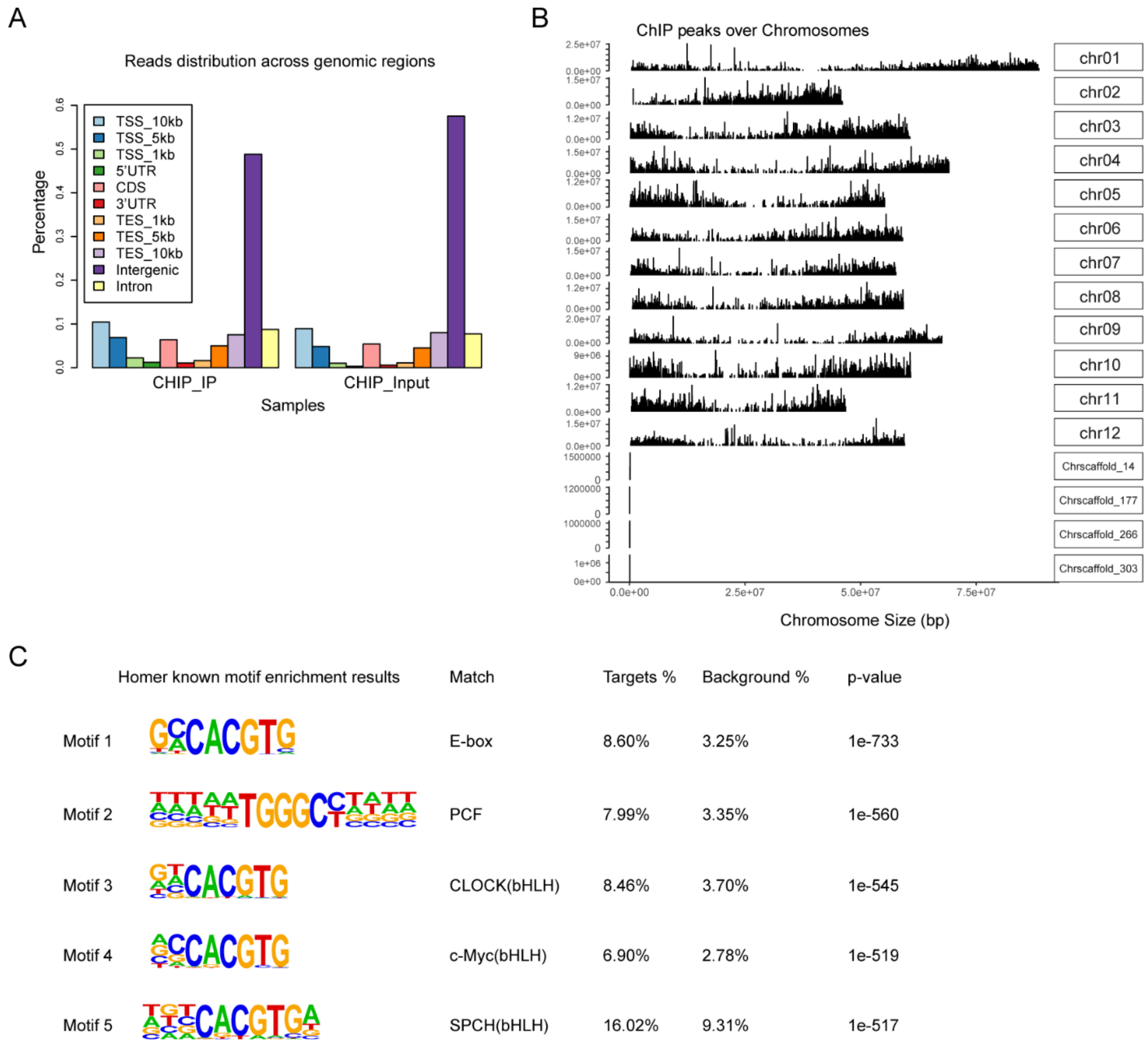

Supplemental Figure S5. Analysis StABL1 binding sites in potato genome. (A) Percentage of reads distribution across different genomic regions. TSS_10 kb, TSS_5 kb and TSS_1 kb: 10 kb, 5 kb and 1 kb upstream of TSS (Transcription start site), respectively. 5'UTR and 3'UTR: 5' and 3' untranslated region. CDS: coding sequence. TES_10 kb, TES_5 kb and TES_1 kb: 10 kb, 5 kb and 1 kb downstream of TES (Transcription start site), respectively. Intergenic: region from 1000 bp down stream of TES (transcription end site) to 2000 bp upstream of TSS. (B) StABL1 binding peaks distribution across different chromosomes. (C) HOMER known motif enrichment analyses of StABL1 binding peaks. The top 5 significantly enriched binding motifs and their matched transcription factor family were presented.

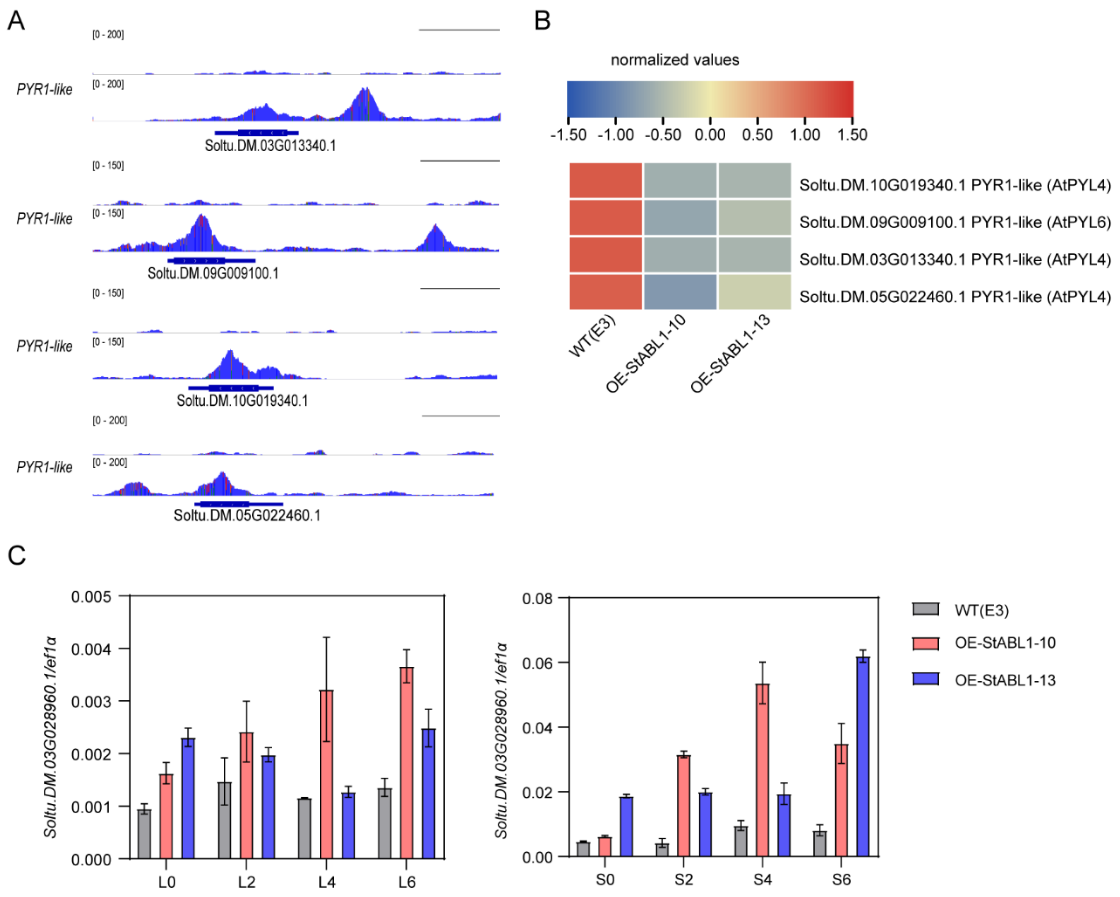

Supplemental Figure S6. Expression analysis of *StABL1*-targeted *PYR1-like* and *AGAMOUS-like* genes. (A) StABL1 binding profiles to putative ABA receptors *PYR1-like* genes visualized with the Integrative Genomics Viewer (IGV). Scale bar: 1 kb. (B) Heatmap showing the normalized expression levels of *PYR1-like* genes. Genes are represented with their gene ID, annotation, and closest *Arabidopsis* homolog. Data represent the mean of three CPM (count per million) values in RNA seq. (C) Time course of *AGAMOUS-like* gene Soltu.DM.03G028960.1 relative expression in wild type E3 and *StABL1*-overexpressing plant leaves and stolon. L0, L2, L4 and L6 indicated leaves sampled at 0, 2, 4 and 6 days after transferred to short day, respectively. S0, S2, S4 and S6 indicated stolon tips sampled at 0, 2, 4 and 6 days after transferred to short day, respectively. Data are presented as mean ± SD. n=3.

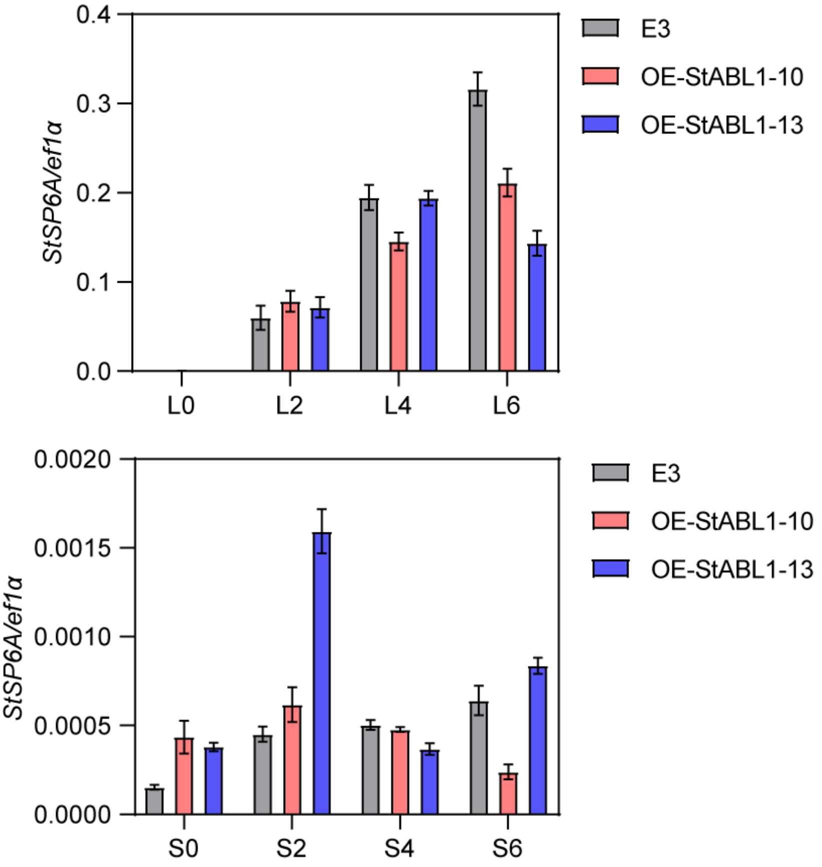

Supplemental Figure S7. Expression analysis of *StSP6A* in wild type E3 and *StABL1*-overexpressing plant leaves and stolon. (A) Time course of *StSP6A* relative expression in wild type E3 and *StABL1*-overexpressing plant leaves and stolon. Sample is same as that in Figure 6B. Data are presented as mean ± SD. n=3.

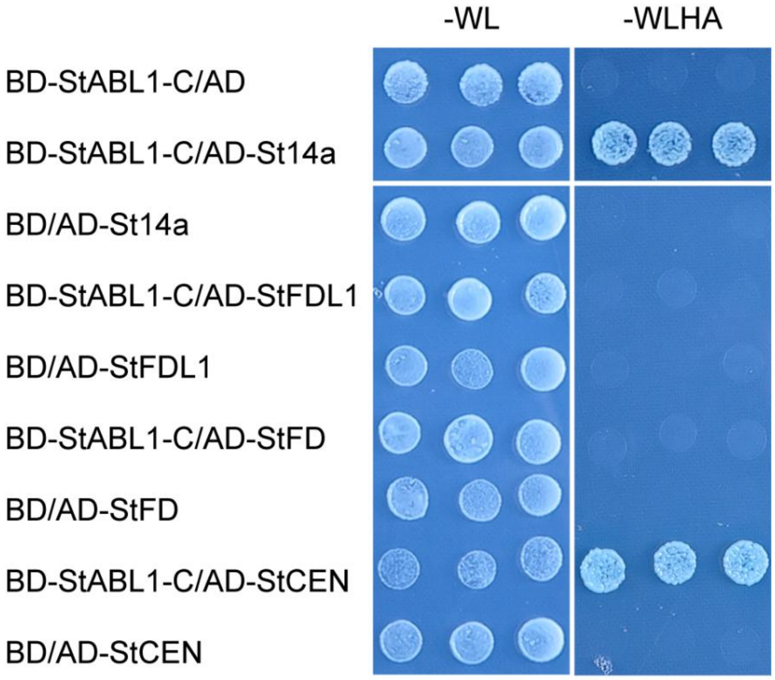

Supplemental Figure S8. Yeast two-hybrid assays demonstrating the interaction between StABL1 and StCEN, StFD and StFDL1 proteins. -WL (medium without tryptophan and leucine); -WLHA (medium without tryptophan, leucine, histidine and adenine). BD (pGBKT7) and AD (pGADT7) are the bait and prey vectors, respectively.

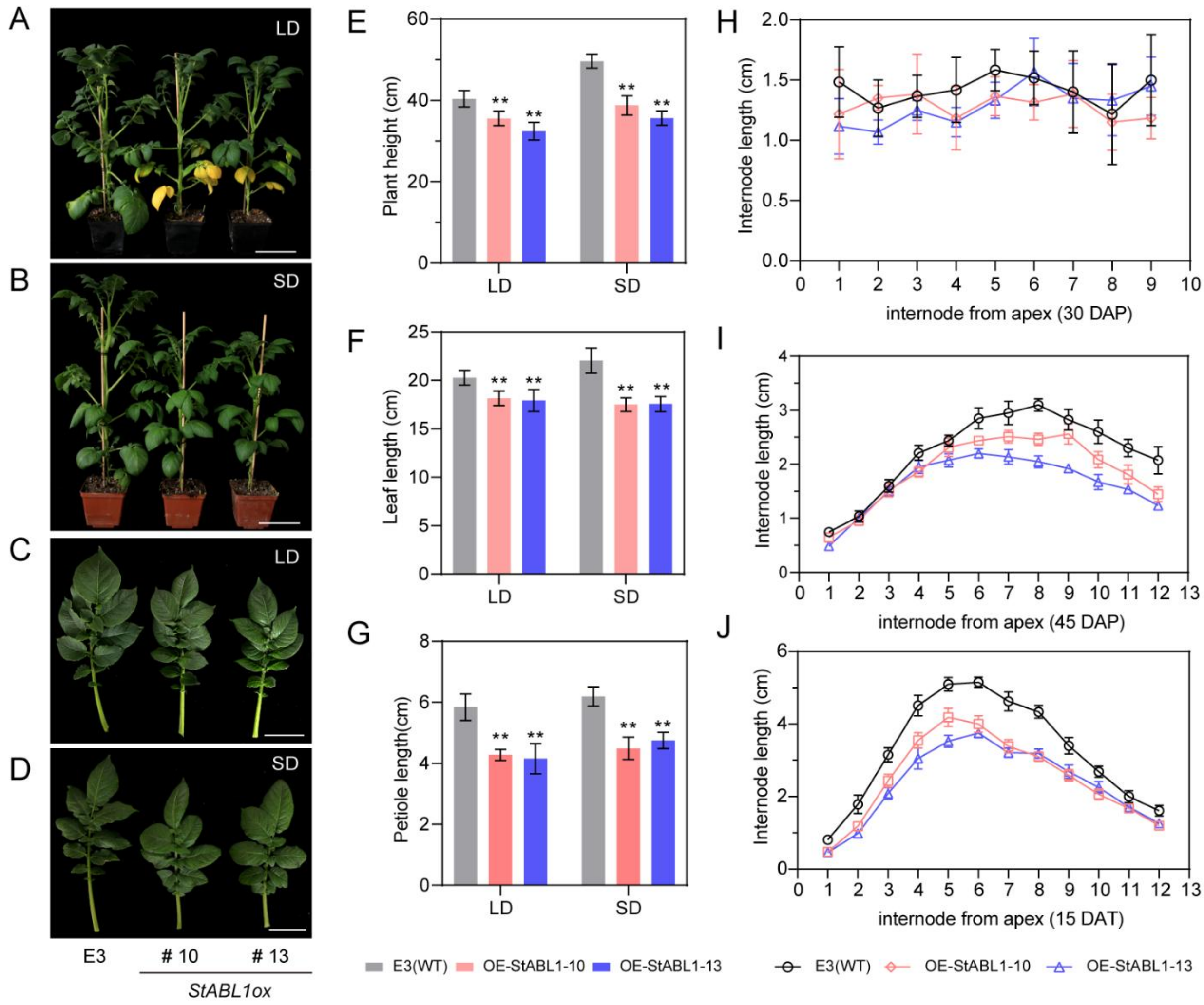

Supplemental Figure S9. Physiological responses to short day in wild type E3 and *StABL1*-overexpressing plants. (A-D) Representative photographs of wild type E3 and *StABL1*-overexpressing plants under long day for 45 days. Plant height (A), leaf length (C); and 15 days in short day after grown in long day for 30 days. Plant height (B), leaf length (D). scale bar in (A-B) represent 10 cm; scale bar in (C-D) represent 5 cm. (E-G) Plant height (E), leaf length (F) and petiole length (G) of wild type E3 and *StABL1*-overexpressing plants under long day (LD) for 45 days. and 15 days in short day (SD) after grown in long day for 30 days, respectively. Data are presented as mean ± SD (n = 8). The asterisks indicate a statistically significant difference (Student's t-test, *P < 0.05, **P < 0.01). (H-J) Internode length of E3 wild

type and *StABL1*-overexpressing plants measured at 30 days after planted (DAP) in long day (H), grown in long day for 45 days (I), and in short day for 15 days after grown in long day for 30 days (J). Data are presented as mean ± SD.

Supplemental Table S1. List of cloning primers used in this study

Primer name	Sequence (5′-3′)	Target gene	Amplicon (bp)	vector	Usage
BK-SP6A-EcoRI-F	ATGGCCATGGAGGCCGAATTCATGCCTAGAGTTGATCCATTGAT	Soltu.DM.05G026370.1	564	PGBKT7	Y2H assay
BK-SP6A-SalI-R	ATGCGGCCGCTGCAGGTCGACTTATGCGCGACGTCCTCC	Soltu.DM.05G026370.1	564	PGBKT7	Y2H assay
SP6A(F99A)-F	CAGCAACTACAAATACAAGCGCGGGAAATGAAG	Soltu.DM.05G026370.1		PGBKT7	PCR-directed mutagenesis
SP6A(F99A)-R	GCTTGTATTTGTAGTTGCTG	Soltu.DM.05G026370.1		PGBKT7	PCR-directed mutagenesis
BK-SP5G-EcoRI-F	ATGGCCATGGAGGCCGAATTCATGCCAAGAGATCCTCTAATAGT	Soltu.DM.05G024030.1	570	PGBKT7	Y2H assay
BK-SP5G-SaLI-R	ATGCGGCCGCTGCAGGTCGACTTATAGGCGACGATCACCG	Soltu.DM.05G024030.1	570	PGBKT7	Y2H assay
SP5G(F98A)-F	CAGCAACCACAGGGGCAACCGCGGGCAATGAAG	Soltu.DM.05G024030.1		PGBKT7	PCR-directed mutagenesis
SP5G(F98A)-R	GGTTGCCCCTGTGGTTGCTG	Soltu.DM.05G024030.1		PGBKT7	PCR-directed mutagenesis
BK-StABL1-EcoRI-F	ATGGCCATGGAGGCCGAATTCATGGGATCTCAGGGTGGTG	Soltu.DM.10G025990.1	1086	PGBKT7	Y2H assay
BK-StABL1-SaLI-R	ATGCGGCCGCTGCAGGTCGACTTAGACGGGCGCGGAG	Soltu.DM.10G025990.1	1086	PGBKT7	Y2H assay
StABL1(S344A)-R	ATGCGGCCGCTGCAGGTCGACTTAGACGGGCGCCGCGCTTGTTCTG	Soltu.DM.10G025990.1		PGBKT7	PCR-directed mutagenesis
StABL1(S344E)-R	ATGCGGCCGCTGCAGGTCGACTTAGACGGGCGCCTCGCTTGTTCTG	Soltu.DM.10G025990.1		PGBKT7	PCR-directed mutagenesis
BK-StABL1-N-R	ATGCGGCCGCTGCAGGTCGACTTAAACAGTCTTCTCAACCACATC	Soltu.DM.10G025990.1		PGBKT7	Y2H assay
BK-StABL1-bZIP-F	ATGGCCATGGAGGCCGAATTCGAAAGGAGGCAAAAGAGGAT	Soltu.DM.10G025990.1		PGBKT7	Y2H assay
BK-StABL1-C-F	ATGGCCATGGAGGCCGAATTCATGTCGACTCCGGGGAAGAAAAGTT	Soltu.DM.10G025990.1		PGBKT7	Y2H assay
BK-StABL1-bZIP-R	ATGCGGCCGCTGCAGGTCGACTTACACCTGCTCTATCTCCTTCT	Soltu.DM.10G025990.1		PGBKT7	Y2H assay
BD-EcoRI-SP3D-F	ATGGCCATGGAGGCCGAATTCATGCCTAGAGAACGCGATCCTCTC	Soltu.DM.03G011110.1	576	PGBKT7	Y2H assay
BD-SalI-SP3D-R	ATGCGGCCGCTGCAGGTCGACTCAATCAGCAGACCTTCTACGTCCA	Soltu.DM.03G011110.1	576	PGBKT7	Y2H assay
AD-SP3D-EcoRI-F	GCCATGGAGGCCAGTGAATTCATGCCTAGAGAACGCGATCCTCTC	Soltu.DM.03G011110.1	576	PGADT7	Y2H assay
AD-SP3D-BamHI-R	CAGCTCGAGCTCGATGGATCCTCAATCAGCAGACCTTCTACGTCCACCA	Soltu.DM.03G011110.1	576	PGADT7	Y2H assay
AD-SP6A-EcoRI-F	GCCATGGAGGCCAGTGAATTCATGCCTAGAGTTGATCCATTGAT	Soltu.DM.05G026370.1	565	PGADT7	Y2H assay
AD-SP6A-BamHI-R	GCAGCTCGAGCTCGATGGATCCTTATGCGCGACGTCCTCC	Soltu.DM.05G026370.1	565	PGADT7	Y2H assay
SP6A(R60K)-F	AAGATCGTCCCCTCCAATATGAACC	Soltu.DM.05G026370.1		PGADT7	PCR-directed mutagenesis
SP6A(R60K)-R	ATATTGGAGGGGACGATCTTAAAAACTTTTACA	Soltu.DM.05G026370.1		PGADT7	PCR-directed mutagenesis
SP6A(P92L)-F	GATATCTGTGACCAGCCAATGTAG	Soltu.DM.05G026370.1		PGADT7	PCR-directed mutagenesis
SP6A(P92L)-R	ATTGGCTGGTCACAGATATCTTGGCAACTACAAA	Soltu.DM.05G026370.1		PGADT7	PCR-directed mutagenesis
AD-St14a-EcoRI-F	GCCATGGAGGCCAGTGAATTCATGGAGAAGGAAAGAGAGAAACAG	Soltu.DM.04G029780.1	801	PGADT7	Y2H assay
AD-St14a-BamHI-R	CAGCTCGAGCTCGATGGATCCCTAGTTCTCTCCCTGGCG	Soltu.DM.04G029780.1	801	PGADT7	Y2H assay
AD-St14f-EcoRI-F	GCCATGGAGGCCAGTGAATTCATGGCGCGTGAGGAGAAT	Soltu.DM.12G006890.1	834	PGADT7	Y2H assay
AD-EcoRI-StFDL1-F	GCCATGGAGGCCAGTGAATTCATGTGGTCATCAAGTAATGAAGA	Soltu.DM.02G005680.1	726	PGADT7	Y2H assay
AD-BamHI-StFDL1-R	CAGCTCGAGCTCGATGGATCCTCAAAATGGGGCCGTTGATGT	Soltu.DM.02G005680.1	726	PGADT7	Y2H assay
AD-StFD-EcoRI-F	GCCATGGAGGCCAGTGAATTCATGTGGTCATCAAGCAGGTCT	Soltu.DM.02G023460.1	549	PGADT7	Y2H assay
AD-StFD-BamHI-R	CAGCTCGAGCTCGATGGATCCTCAAAATGGAGCGGTTGACGTC	Soltu.DM.02G023460.1	549	PGADT7	Y2H assay
AD-CEN-EcoRI-F	GCCATGGAGGCCAGTGAATTCATGTCTTCTAGAGGTACTTGTGAAC	Soltu.DM.03G017110.1	570	PGADT7	Y2H assay
AD-CEN-EcoRI-R	CAGCTCGAGCTCGATGGATCCTCATCTTCTTCTAGCTGCAGTTTC	Soltu.DM.03G017110.1	570	PGADT7	Y2H assay
AD-St14f-BamHI-R	CAGCTCGAGCTCGATGGATCCCAAATGATAATCTGAGGACCAGTTCC	Soltu.DM.12G006890.1	834	PGADT7	Y2H assay
HA-GFP-StuI-F	AGATTATGCTGATTACGCCGAGATGGTGAGCAAGGGCGAG	GFP	759	pH7LIC7.0-N-HA	Co-IP
HA-GFP-StuI-R	GGATATCATTAGGGAAGAGGCTTGTACAGCTCGTCCATGC	GFP	759	pH7LIC7.0-N-HA	Co-IP
GFP-Bsp1407I-StABL1-R	TATCACCACTTTGTACATTAGACGGGCGCGGAGC	Soltu.DM.10G025990.1	1080	PB7WGF2	Subcellular localization/Co-IP
GFP-Bsp1407I-StABL1-R	CATGGACGAGCTGTACAAGATGGGATCTCAGGGTGGTG	Soltu.DM.10G025990.1	1080	PB7WGF2	Subcellular localization/Co-IP
HA-StuI-St14a-F	AGATTATGCTGATTACGCCGAGATGGAGAAGGAAAGAGAGAAACAG	Soltu.DM.04G029780.1	801	pH7LIC7.0-N-HA	Co-IP
HA-StuI-St14a-R	GGATATCATTAGGGAAGAGGCTAGTTCTCTCCCTGGCG	Soltu.DM.04G029780.1	801	pH7LIC7.0-N-HA	Co-IP
HA-StuI-St14f-F	AGATTATGCTGATTACGCCGAGATGGCGCGTGAGGAGAAT	Soltu.DM.12G006890.1	807	pH7LIC7.0-N-HA	Co-IP
HA-StuI-St14f-R	GGATATCATTAGGGAAGAGGTCACTGTTGTTCATTGTCG	Soltu.DM.12G006890.1	807	pH7LIC7.0-N-HA	Co-IP
SP6A-RFP-SpeI-F	CCTGCAGGCGGCCGCACTAGTATGCCTAGAGTTGATCCATTGAT	Soltu.DM.05G026370.1	561	PB7WGR2	Subcellular localization
SP6A-RFP-SpeI-R	GACGTCCTCGGAGGAGGCCATTGCGCGACGTCCTCCA	Soltu.DM.05G026370.1	561	PB7WGR2	Subcellular localization
YN-StABL1-BamHI-F	CTGAGGAGGATCTTGGATCCATGGGATCTCAGGGTGGTG	Soltu.DM.10G025990.1	1086	NYFP	BiFC
YN-StABL1-SalI-R	TACCGAATTCACTAGTGTCGACTTAGACGGGCGCGGAGCTTGTTCTG	Soltu.DM.10G025990.1	1086	NYFP	BiFC
YN-SP6A-BamHI-F	CTGAGGAGGATCTTGGATCCATGCCTAGAGTTGATCCATTGAT	Soltu.DM.05G026370.1	562	NYFP	BiFC
YN-SP6A-SalI-R	CCGAATTCACTAGTGTCGACTTATGCGCGACGTCCTCC	Soltu.DM.05G026370.1	562	NYFP	BiFC
YN-SP3D-BamHI-F	CTGAGGAGGATCTTGGATCCATGCCTAGAGAACGCGATCCTCTC	Soltu.DM.03G011110.1	574	NYFP	BiFC
YN-SP3D-SalI-R	CCGAATTCACTAGTGTCGACTCAATCAGCAGACCTTCTACGTCCA	Soltu.DM.03G011110.1	574	NYFP	BiFC
YC-StABL1-BamHI-F	TTCCAGATTACGCTGGATCCATGGGATCTCAGGGTGGTG	Soltu.DM.10G025990.1	1086	CYFP	BiFC
YC-StABL1-SalI-R	CCGAATTCACTAGTGTCGACTTAGACGGGCGCGGAG	Soltu.DM.10G025990.1	1086	CYFP	BiFC
YC-St14a-BamHI-F	TTCCAGATTACGCTGGATCCATGGAGAAGGAAAGAGAGAAACAG	Soltu.DM.04G029780.1	799	CYFP	BiFC
YC-St14a-SalI-R	CCGAATTCACTAGTGTCGACCTAGTTCTCTCCCTGGCG	Soltu.DM.04G029780.1	799	CYFP	BiFC
Ri-XbaI-StABL1-F	GATAAGCTTGGATCCTCTAGAATGAGCTTGAGCTTGAGCT	Soltu.DM.10G025990.1	441	pHELLSGATE 8	RNAi
Ri-XbaI-StABL1-R	TCATTAAAGCAGGACTCTAGAATTTGACTCTTCCCTTTTCTGGG	Soltu.DM.10G025990.1	441	pHELLSGATE 8	RNAi
Ri-XhoI-StABL1-F	TTTGGAGAGGACACGCTCGAGATTTGACTCTTCCCTTTTCTGG	Soltu.DM.10G025990.1	441	pHELLSGATE 8	RNAi
Ri-XhoI-StABL1-R	TGGGGTACCGAATTCCTCGAGATGAGCTTGAGCTTGAGCT	Soltu.DM.10G025990.1	441	pHELLSGATE 8	RNAi
GFP-StABL1-BamHI-F	CTAGAGGATCCATGGTGAGCAAGGGCG	Soltu.DM.10G025990.1	1800	PBI121	overexpression
GFP-StABL1-SacI-R	AATTCGAGCTCTATCACCACTTTGTACATTAGACGG	Soltu.DM.10G025990.1	1800	PBI121	overexpression

Supplemental Table S2. List of qRT-PCR primers used in this study

Primer name	Sequence (5´-3´)	Target gene	Amplicon (bp)	Usage
qRT-StABL1-F	GAACTGGAGAACAAGGTTTCAC	Soltu.DM.10G025990.1	196	qPCR
qRT-StABL1-R	GACATAACGAACACTACGCATC	Soltu.DM.10G025990.1	196	qPCR
StABL1-TG-F	GCAGGCTTACACCCATGAAC	Soltu.DM.10G025990.1	104	qPCR(overexpression check)
StABL1-TG-R	GAACACTCGGTAACACCTGC	Soltu.DM.10G025990.1	104	qPCR(overexpression check)
qRT-StGA2ox1 -F	AGGCACAGAGTGATCGCAGAT	Soltu.DM.02G013470.1	65	qPCR
qRT-StGA2ox1 -R	TGGTGGCCCTCCAAAGTAAA	Soltu.DM.02G013470.1	65	qPCR
qRT-StGA20ox-F	GCTAGCTTTACTGGTATCCAATGT	Soltu.DM.03G016400.1	111	qPCR
qRT-StGA20ox1-R	TCATAATGTGCTTGAGACGCC	Soltu.DM.03G016400.1	111	qPCR
RTef1α- F	ATTGGAAACGGATATGCTCCA	Soltu.DM.06G005620.1	101	qPCR
RTef1α -R	TCCTTACCTGAACGCCTGTCA	Soltu.DM.06G005620.1	101	qPCR
qRT-SP6A-F	GACGATCTTCGCAACTTTTACA	Soltu.DM.05G026370.1	75	qPCR
qRT-SP6A-R	CCTCAAGTTAGGGTCGCTTG	Soltu.DM.05G026370.1	75	qPCR
qRT-28960-F	CGTGGGAGCAACAGAGTCAT	Soltu.DM.03G028960.1	114	qPCR
qRT-28960-R	GCTTCCCCCATGTGAAGGTT	Soltu.DM.03G028960.1	114	qPCR
